# Supplementary material for: Functional Janus structured liquids and aerogels
Source: Nat Commun. 2023 Nov 28;14:7811. doi: 10.1038/s41467-023-43319-7 (PMC10684591; doi:10.1038/s41467-023-43319-7)
Supplement: Supplementary file 1 — Supplementary Information [file 41467_2023_43319_MOESM1_ESM.pdf]

Functional Janus Structured Liquids and Aerogels

Ahmadreza Ghaffarkhah[1,2,10], Seyyed Alireza Hashemi[1,10], Farhad Ahmadijokani[1,2], Milad Goodarzi[1], Hossein Riazi[3], Sameer E. Mhatre[2], Orysia Zaremba[4], Orlando J. Rojas[2], Masoud Soroush[3], Thomas P. Russell[5,6,7*], Stefan Wuttke[4,8*], Milad Kamkar[9*], and Mohammad Arjmand[1*]

[1] Nanomaterials and Polymer Nanocomposites Laboratory, School of Engineering, University of British Columbia, Kelowna, BC, V1V 1V7, Canada

[2] Bioproducts Institute, Department of Chemical & Biological Engineering, Department of Chemistry and Department of Wood Science, 2360 East Mall, The University of British Columbia, Vancouver, BC V6T 1Z3, Canada

[3] Department of Chemical and Biological Engineering, Drexel University, Philadelphia, PA 19104, USA

[4] Basque Centre for Materials, Applications & Nanostructures (BCMaterials), Bld. Martina Casiano, 3rd. Floor UPV/EHU Science Park Barrio Sarriena s/n, Leioa, 48940 Spain

[5] Polymer Science and Engineering Department, University of Massachusetts Amherst, 120 Governors Drive, Amherst, MA, 01003, USA

[6] Materials Sciences Division, Lawrence Berkeley National Laboratory, 1 Cyclotron Road, Berkeley, CA, 94720, USA

[7] Advanced Institute for Materials Research (WPI-AIMR), Tohoku University, 2-1-1 Katahira, Aoba, Sendai 980-8577, Japan

[8] IKERBASQUE, Basque Foundation for Science, Bilbao, 48013, Spain

[9] Department of Chemical Engineering, Waterloo Institute for Nanotechnology, University of Waterloo, 200 University Avenue West, Waterloo, Ontario N2L 3G1, Canada

[10] These authors contributed equally to this work.

Corresponding authors: mohammad.arjmand@ubc.ca, milad.kamkar@uwaterloo.ca, stefan.wuttke@bcmaterials.net, russell@mail.pse.umass.edu

Contents

1. Experimental 3

1.1. Materials 3

1.2. Methods 4

1.2.1. Synthesis of well-exfoliated GO 4

1.2.2. Synthesis of magnetic GO (mGO) 5

1.2.3. Synthesis of $Ti_3C_2T_x$ flakes 5

1.2.4. Fabrication of structured liquids and aerogels 6

1.2.5. Rationalization of Janus design: Controllable functionality and liquid templating concept 9

1.2.6. Controllability of Janus liquids: Tuning composition and functionality for tailored constructs 9

1.3. General characterization 10

2. Characterization of the synthesized nanomaterial 13

3. Shielding setup and background concept 17

3.1. Conceptual mechanism of EMI shielding 18

3.2. EMI shielding parameters and formulations 20

3.3. Shielding effectiveness and logarithmic parameters 22

4. Figures 27

5. Tables 58

Data declaration and availability 61

References 62

1. Experimental

1.1. Materials

Herein, active nanomaterials were synthesized using analytical-grade chemical compounds. For the synthesis of graphene oxide (GO), utilized chemical reagents such as highly-concentrated sulfuric acid (H_2SO_4 95-98%), orto-phosphoric acid (H_3PO_4 85%), potassium permanganate ($KMnO_4$), hydrogen peroxide (H_2O_2 30%), anhydrous ethanol (CH_3CH_2OH with ≤0.003% H_2O), and hydrochloric acid (HCl 37%) were supplied by Sigma-Aldrich. Additionally, high-quality graphite flakes as the source for obtaining the well-exfoliated GO were supplied by Asbury Carbons.

For the synthesis of magnetic nanoparticles, viz., Fe_3O_4 nanoparticles, analytical grade iron (II) chloride tetrahydrate ($FeCl_2.4H_2O$), iron (III) chloride hexahydrate ($FeCl_3.6H_2O$), and ammonium hydroxide (NH_4OH 28-30%) were used (supplied by Sigma-Aldrich). For the fabrication of multi-layer $Ti_3C_2T_x$ flakes, i.e., $Ti_3C_2T_x$, analytical grade reagents such as hydrofluoric acid (HF) and HCl (41-58 %) were supplied by Fisher Scientific Co. The Titanium Aluminum Carbide (Ti_3AlC_2), viz., MAX phase, was supplied by Anasori's lab at Indiana University Purdue University Indianapolis. The lithium chloride (LiCl 99.98%) was used for the delamination of multi-layer $Ti_3C_2T_x$ flakes and supplied by the Alfa Aser. Throughout the synthesis processes, ultra-pure deionized water with a resistivity of 18.2 MΩ was utilized (ELGA model MEDICA EDI 15/30). A mixture of hexane (97%) and PSS-[3-(2-Aminoethyl)amino] propyl-heptaisobutyl substituted (POSS) was used in liquid streaming, both supplied by Sigma-Aldrich.

1.2. Methods

1.2.1. Synthesis of well-exfoliated GO

GO was synthesized according to the reported modified Hummers Method by Marcano et al. [1] with slight modifications according to our previous procedures [2, 3, 4, 5, 6, 7]. Briefly, highly concentrated sulfuric acid (H_2SO_4 95-98%) and orto-phosphoric acid (H_3PO_4 85%) were added together with a ratio of 9:1 (360 mL:40 mL), followed by stirring at 300 rpm for 10 min. Next, 3 g of graphite powder was added to the solution and stirred for 30 min. The temperature of the acidic/graphitic suspension was reduced to about ∼0-5 °C using an ice bath, followed by the slight addition of 18 g potassium permanganate ($KMnO_4$) in the time span of 30 min. The resulting mixture was stirred for a further 30 min, and thence the suspension temperature was increased with a slight ramp to 50 °C. The reaction was allowed to stir for 18 h under the same temperature and thence terminated via a mixture of 800 mL ultrapure deionized water (18.2 MΩ resistivity) and 5 mL hydrogen peroxide (H_2O_2, 30%). Then, the resulting exfoliated GO flakes underwent purification and separation protocols. Accordingly, the resulting product was washed several times with water, 30 vol% HCl, and ethanol to remove the impurities. The resulting mixture was diluted to a concentration of 2 mg/ml and ultrasonicated within an ultrasonic bath for 30 min at moderate power while maintaining the temperature limit at 30 °C. The unexfoliated few-layer graphite oxide flakes were separated from well-exfoliated GO using centrifugation at 3500 rpm (relative centrifugal force (RCF) = 1575) for 1 h, followed by the supernatant collection as the main purified product. The obtained GO flakes were concentrated via centrifugation at 11000 rpm (RCF = 15557) for 1 h. The final product was kept in a sealed container for further experiments and sample preparations.

1.2.2. Synthesis of magnetic GO (mGO)

For the decoration of GO with magnetic nanoparticles (Fe_3O_4), first, the magnetic nanoparticles were synthesized and thence immobilized on the surface of GO through the ultrasonic assembly. Briefly, the magnetic nanoparticles were synthesized via co-precipitation of iron (II) chloride tetrahydrate ($FeCl_2.4H_2O$) and iron (III) chloride hexahydrate ($FeCl_3.6H_2O$). Accordingly, $FeCl_2·4H_2O$ and $FeCl_3·6H_2O$ were added into 100 mL of ultrapure deionized water in a molar ratio of 2:1. The mixture was vigorously stirred at room temperature under a nitrogen (N_2) atmosphere. Afterward, ammonia solution was dropwise added to the resulting suspension till reaching pH 9. The suspension was stirred for 1h at 60 °C until forming Fe_3O_4 nanoparticles. Next, the precipitated magnetic nanoparticles were washed several times with ultrapure deionized water and separated via a strong magnet. The resulting Fe_3O_4 nanoparticles were dried at 60 °C overnight. For the immobilization of Fe_3O_4 on the GO, 10 mL of the stock solution of GO (concentration of 10 mg/ml) was dispersed in 25 mL of deionized water; meanwhile, 100 mg of the magnetite nanoparticles were dispersed in 15 mL of deionized water using ultrasonication and subsequent stirring. The GO solution was placed under a probe sonicator, and the stable Fe_3O_4 suspension was dropwise added to it. The resulting mixture was stirred at 50 °C overnight and thence concentrated using centrifugation at 11000 rpm (RCF = 15557) for further experiments.

1.2.3. Synthesis of $Ti_3C_2T_x$ flakes

The well-exfoliated $Ti_3C_2T_x$ flakes were synthesized according to our previous protocols [8]. For the synthesis of multi-layer $Ti_3C_2T_x$ out of the MAX phase (Titanium Aluminum Carbide (Ti_3AlC_2)), first, 2 g of highly purified Ti_3AlC_2 MAX phase with an average particle size of <75 µm was slightly added to a solution composed of 2 mL HF (29 M), 6 mL HCl (12 M), and 12 mL ultrapure deionized water over the course of 5 min. The reaction medium was stirred for 24 h at ambient

temperature, and once completed, the highly acidic medium was centrifuged at 3500 rpm (RCF = 1575) for 5 min to remove the by-products, followed by washing with deionized water. The washing cycle continued until the supernatant's pH reached the value of 6. The as-synthesized product contains a few layers of $Ti_3C_2T_x$ flakes and must undergo a delamination process. For this aim, 2 g LiCl was added to the stock solution of the few-layer $Ti_3C_2T_x$ flakes (40 mL stock solution), followed by stirring at 350 rpm overnight. Next, the suspension was applied to severe shaking to deaminate multi-layer $Ti_3C_2T_x$ flakes, followed by subsequent separation of unreacted/non-delaminated products via centrifugation at 3500 rpm (RCF = 1575) for 5 min. The obtained product was re-dispersed in water and concentrated for further use.

1.2.4. Fabrication of structured liquids and aerogels

1.2.4.1 $Ti_3C_2T_x$ beads

$Ti_3C_2T_x$ aqueous suspensions with a concentration of 5-10 mg/ml were loaded into a plastic syringe and extruded into the hexane containing 1 mg/ml POSS using a 3D Bioprinter (Allevi, USA). In the case of the 200 µm (ID) dispensing needle, the extrusion pressure was set at 10-15 psi. Upon injection, the aqueous jet of $Ti_3C_2T_x$ breaks into discrete droplets, forming what we have called liquid beads. Such a structure can also be achieved with the aid of a simple compressor or extrusion of the aqueous phase by hand. $Ti_3C_2T_x$ liquid beads were used for aerogel fabrication. To achieve this, samples were kept in a freezer overnight, and then the hexane phase was removed. Finally, the frozen aqueous phase was freeze-dried for two days to achieve MXene aerogel beads.

1.2.4.2 GO worm-like structures

GO-liquid threads with a worm-like morphology were achieved by streaming a 5-10 mg/ml GO aqueous suspension into hexane containing POSS. Here, the rapid co-assembly of GO and POSS at the interface generates an elastic interfacial skin around the extruded thread that prevents the

breakup of an aqueous jet into droplets, i.e., suppressing Plateau-Rayleigh instabilities. In the case of the 200 µm (ID) dispensing needle, the extrusion pressure was set at 15-30 psi. Such a structure can be achieved with the aid of a 3D Bioprinter, a simple compressor, or extrusion of the aqueous phase by hand. GO worm-like aerogels were fabricated through a similar procedure explained in section 1.2.4.1.

1.2.4.3 $Ti_3C_2T_x$/GO worm-like structures

Deciphering the inherent characteristics of GO and $Ti_3C_2T_x$, we also designed hybrid aqueous systems containing both NPs for liquid streaming. Here, aqueous tubules like that of pure GO were achieved for $Ti_3C_2T_x$/GO inks. Unless otherwise specified, we employed a gauge 27 dispensing needle with an inner diameter of 200 µm, along with an extrusion pressure of 10-15 psi for the fabrication of these structures. Such a structure can be achieved with the aid of a 3D Bioprinter, a simple compressor, or extrusion of the aqueous phase by hand. $Ti_3C_2T_x$/GO worm-like aerogels were fabricated through a similar procedure explained in section 1.2.4.1. Four types of $Ti_3C_2T_x$/GO inks based on the different ratios of NPs and different solid contents are prepared:

- $Ti_3C_2T_x$/GO inks with a total solid concentration of 10 mg/ml containing 20 and 80 wt% of GO (weight of GO compared to the total weight of NPs).
- $Ti_3C_2T_x$/GO inks with a total solid concentration of 5 mg/ml containing 20 and 80 wt% of GO (weight of GO compared to the total weight of NPs).

1.2.4.3 Janus liquids and aerogels

Janus liquid threads were fabricated by merging two streams of structured liquids in the hexane/POSS domain. In the case of magnetic/conductive structures, two different streams of 10 mg/ml $Ti_3C_2T_x$/GO (20 wt% GO) and 10 mg/ml mGO/GO (50 wt% GO) were merged. However,

for the case of conductive/non-responsive constructs, two streams of 10 mg/ml $Ti_3C_2T_x$/GO (20 wt% GO) and 10 mg/ml GO were merged to form the Janus constructs. The injection heads were placed in a way that the two streams collide and merge into Janus liquids threads, as shown in Figure 3a-b. In the case of the 200 µm (ID) dispensing needle, the extrusion pressure was set at 10-15 psi for $Ti_3C_2T_x$/GO and 15-20 psi for mGO/GO or pure GO inks.

Interestingly, the ratio of opposing parts in the Janus liquid threads can be controlled by tuning the streaming parameters. For instance, using different needles for each ink, i.e., 200 µm for $Ti_3C_2T_x$/GO and 400 µm for mGO/GO, we fabricated Janus structures in which the magnetic parts' volume is larger than that containing $Ti_3C_2T_x$. The extrusion process involved adjusting the pressure of $Ti_3C_2T_x$/GO from 20 to 15 psi. Janus structures can be achieved with the aid of a 3D Bioprinter that has two injection heads, a simple compressor, or extrusion of aqueous phase by hand. Janus aerogels were fabricated through a similar procedure explained in section 1.2.4.1.

For magnetic/conductive Janus EMI shields, 6 ml of conductive ink [10 mg/ml $Ti_3C_2T_x$/GO (20 wt% GO)] and 8 ml of magnetic ink [10 mg/ml mGO/GO (50 wt% GO)] were used. Both inks were extruded through 200 µm (ID) dispensing needles. At the beginning of the process, only mGO/GO ink was extruded for around 3s (almost 1 ml of mGO/GO ink). After that, both conductive and magnetic inks were simultaneously extruded and merged to form Janus liquid treads. The volume of magnetic and conductive inks was selected in a way that the conductive ink finished slightly sooner than the magnetic ink, and therefore, the top surface of the sculpted structure mainly covers with magnetic filaments. After freeze-drying, the prepared magnetic/conductive aerogels with a thickness of ~5 mm were used as free-standing EMI absorbers.

We also developed a unique EMI trap using the magnetic/conductive Janus aerogels. This structure consists of a 3mm Janus aerogel covered with 0.05 mm conductive copper tape from one side. The Janus aerogel of this shield is designed in a way that the magnetic parts' volume is larger than the conductive domain, i.e., the mGO/GO (50 wt% GO) and $Ti_3C_2T_x$/GO (20 wt% GO) inks were extruded from 400 and 200 µm nozzle, respectively. In this case, the impedance mismatch between this Janus structure and free space is even lower than the Janus aerogels prepared in the previous section, allowing the incident waves to penetrate the structure with minimum reflection.

Janus aerogels of $Ti_3C_2T_x$/GO (20 wt% GO): GO were adopted for pressure sensing. The solid concentration of both inks was 10 mg/ml, and 200 µm (ID) dispensing needles were used for both aqueous suspensions. After freeze-drying, two copper wires were attached to the two sides of the constructs, and the whole structure was covered by double-sided elastomeric tapes. Afterward, a multichannel potentiostat (Metrohm Autolab, the Netherlands) was employed to investigate the current vs. time behavior of the printed sensors at a constant voltage of 1 V. The behavior of the sensor at various compression conditions was evaluated with a tensile apparatus (Instron universal testing machine, USA). Finally, the Janus sensors were employed for real-time human motion and health monitoring. Written consent from all participants was obtained prior to the research.

1.2.5. Rationalization of Janus design: Controllable functionality and liquid templating concept

Janus liquids can be visualized as liquid threads consisting of two distinct sections, i.e., faces, each composed of a specific group of NPs. This duality embodies the essence of Janus, representing a remarkable opportunity to assign distinct functionalities to opposing sides of these structures and independently fine-tune them. This landmark nature of the Janus liquids becomes even more

pronounced when they are used as a liquid template, enabling the development of customized compositions and arrangements for task-oriented aerogels.

The possible uses of Janus aerogels are limitless, especially when a deliberate connection is established between the functionality of the Janus building blocks and the ultimate application. For instance, developing Janus aerogels with non-interfering magnetic/conductive opposing sections is valuable for EMI shielding applications where alternating magnetic/conductive domains of the aerogels create numerous interfaces within a small volume to absorb the electromagnetic waves (see the subsequent discussion in the following sections and Supplementary Information). One other application involves the development of Janus aerogels, where one section of the structure exhibits specific functionality, while the other imparts desired mechanical characteristics, as described in this paper. Overall, this anisotropic integration platform with adjustable functionality has many potential applications where the simultaneous encoding of multiple functionalities in a single construct is highly beneficial, e.g., Janus gas/liquid absorbers, where each section is fine-tuned to deal with a specific type of material, or multi-modal sensors that can accurately and independently sense two different classes of analytes.

1.2.6. Controllability of Janus liquids: Tuning composition and functionality for tailored constructs

Controllability in the fabrication of Janus liquids allows the composition and functionality of the final constructs to be independently tuned. This controllability can be defined through the following means:

- Control over concentration and composition: Janus liquid threads are created by joining two aqueous streams of nanoparticle (NP) dispersions in an apolar domain. This method

allows effective tuning of the concentration and composition of each building block. For example, Figures 2, S9, and S10 demonstrate various conductive liquid threads of $Ti_3C_2T_x$/GO with different compositions and concentrations. The wide range of options for the conductive portion of the Janus structured liquids enables precise control over the composition, influencing the density and electrical conductivity of the subsequent sections in Janus aerogels. The same flexibility applies to the non-responsive portion, where different suspensions of GO with concentrations ranging from 5 to 10 mg/ml were used in liquid streaming, as illustrated in Figures 1a-e and S6.

- Control over the relative ratio of opposing building blocks: By adjusting the streaming parameters, the ratio of opposing parts of the Janus liquid threads can be controlled. For instance, using different needles for each ink, such as a 200 µm needle for $Ti_3C_2T_x$/GO and a 400 µm needle for mGO/GO, allows the fabrication of Janus structures with a larger volume of magnetic parts compared to the volume containing $Ti_3C_2T_x$, as shown in Figures S16 a-d.
- Control over the subsequent functionality: The concept of Janus liquid threads enables the allocation of specific nanomaterials with distinct characteristics to opposite sides, resulting in specific functionalities. In this research, materials with magnetic, non-responsive, or electrically conductive properties were assigned to different regions of Janus constructs, as shown in Figures 3, S15-16, and S21. This concept holds great promise for various applications, such as developing multi-responsive gas sensors or designing Janus structures for the efficient absorption of different classes of pollutants.

1.3. General characterization

The as-developed nanomaterials and aerogels were assessed via diverse analyses to confirm their successful fabrication with the desired quality. The $Ti_3C_2T_x$ flakes were coated on the surface of a silicon wafer, dried at 60 °C, and applied for further analyses. Accordingly, the general characterizations were conducted via Fourier-transform infrared spectroscopy (FTIR) (Nicolet-S20) within the range of 600-4000 cm^{-1}, X-ray diffraction (XRD) (Brucker D8 Advance), and micro-Raman spectroscopy (Brucker Senterra II) equipped with an Olympus U-TV1X-2 microscope. Additionally, the morphological assessments of the developed aerogels were conducted via Field Emission Scanning Electron Microscope (FESEM) (Tescan Mira 3 XMU) equipped with an Oxford Instruments X-Max energy dispersive spectrometer (EDS) detector. The morphology and atomic structure of the GO and $Ti_3C_2T_x$ flakes were assessed via high-resolution transmission electron microscopy (HRTEM) (80-300 Titan LB) equipped with an aberration-corrector (CEOS), operated at 300 kV; images were also acquired using a FirstLight CCD, Gatan. The electrical conductivity of the developed aerogels was measured via a Loresta GP resistivity meter (Mitsubishi Chemical Co. model MCP-T700). The 3D morphology of Janus nanofibers and the interface of filaments with each other were assessed via a 3D laser scanning microscope (Olympus model LEXT OLS5000). X-Ray micro-computed tomography (micro-CT) images of the prepared aerogels were obtained using 3D X-ray microscopes (ZEISS Xradia 520 Versa). The interfacial rheology and dynamic interfacial tension (IFT) tests of aquatic GO or $Ti_3C_2T_x$-based inks were assessed by holding the 5 µL of aquatic droplets of inks' suspensions in the organic hexane phase. Accordingly, the dynamic interfacial tension assessments in the pure hexane were performed in 1 h. This time did not prolong because of the rapid interfacial solidification and loss of sphericity of the drop in the host aquatic media containing POSS and hexane.

2. Characterization of the synthesized nanomaterial

The synthesized 2D flakes, including $Ti_3C_2T_x$, GO, and mGO, were assessed using different characterization techniques. In Figures S1a-b, the X-ray diffractogram of $Ti_3C_2T_x$ flakes heated at 60 °C and 250 °C can be seen, respectively. As depicted in part (a), the well-defined 2θ peaks at 7.91, 16.94, 26.84, 35.09, and 43.76 are attributed to the (002), (004), (006), (008), and (0010) crystalline planes of $Ti_3C_2T_x$, respectively [9]. Heating the $Ti_3C_2T_x$ flakes at 250 °C affected the interlayer spacing of these flakes and led to a slight shift of 2θ peaks to 8.36, 17.36, 26.33, 35.21, and 44.33, respectively.

The micro-Raman spectroscopy provides essential information regarding the quality and functionality of $Ti_3C_2T_x$ flakes. Figure S1c illustrates the Raman spectrum of $Ti_3C_2T_x$ flakes in the range of 100 to 800 cm^{-1}, obtained via the 785 nm laser. The vibration of the $Ti_3C_2T_x$ could be described by the mean of Mulliken symbols as $4E_g+2A_{1g}+4E_u+2A_{2u}$, where E_g and A_{1g} belong to the active modes of Raman, while E_u and A_{2u} are attributed to the active modes of IR. The E_g and A_{1g} correspond to the in-plane and out-of-plane vibrations of carbon and titanium atoms, respectively. On the other hand, active modes of IR, viz., E_u and A_{2u}, originated from the in-plane and out-of-plane vibrations of carbon atoms [10].

Generally speaking, the Raman spectrum of $Ti_3C_2T_x$ can be divided into three main regions, as illustrated in Figure S1c. These regions include the (I) flake region (including a sharp peak at 203 cm^{-1}), which is attributed to the vibration of titanium layers, carbon, and surface functional groups, (II) the T_x region (230 to 470 cm^{-1}), corresponding to the vibration of surface functional groups, viz., O(OH), O_2, and $(OH)_2$, attached to the Ti atoms, and (III) the carbon region (580-730 cm^{-1}), attributed to the in-plane and out-of-plane vibrations of carbon atoms within the structure of

$Ti_3C_2T_x$ [10]. Out of these regions, there is a strong and sharp peak at about 120.5 cm^{-1}, attributed to the resonant peak and responsible for the plasmonic properties of the $Ti_3C_2T_x$ [11, 12].

The flake region mainly consisted of A_{1g} (Ti, C, O) and E_g (Ti, C, O) modes, attributed to the out-of-plane and in-plane vibrations of surface functional groups and Ti atoms within the outer layer along with carbon atoms. This region shows the vibration of flake as the vibration of surface functional groups, titanium and carbon. The Flake region includes the maximum number of atoms from the unit cell of $Ti_3C_2T_x$. Moreover, the presence of water molecules within the structure of $Ti_3C_2T_x$ improves the stiffness of out-of-plane vibrations and enlarges the interlayer spacing. This matter shifts the A_{1g} (C) peak to about 720 cm^{-1}. Such a shift in the A_{1g} (C) peak reveals that the vibrations of carbon layers within the $Ti_3C_2T_x$ flake do not depend on the flake's orientation [10]. These outcomes depict the successful synthesis of $Ti_3C_2T_x$ with high quality and essential functionalities for external applications.

Figure S1d shows the FTIR spectrum of $Ti_3C_2T_x$ flakes. As depicted, the delaminated $Ti_3C_2T_x$ flakes revealed a well-defined FTIR spectrum with the desired functional groups for homogeneous dispersion within the aquatic media. As illustrated, the broad peak at 3349.26 cm^{-1} corresponds to the stretching vibration of hydroxyl (-OH) functional groups. Other appeared peaks at about 2900.41 and 1636.78 cm^{-1} are attributed to the $-CH_2$ and -OH functional groups, respectively. Numerous appeared peaks in area A of Figure S1d correspond to the vibration of different functional groups on the surface of $Ti_3C_2T_x$ flakes, including C-F (1099.70 cm^{-1}), O-H (1395.24 cm^{-1}), C-H (1456.95 cm^{-1}), aromatic ring (1507.09 cm^{-1}), and C=C (1559.16 cm^{-1}), respectively [13, 14]. The outcome of the XRD, micro-Raman spectroscopy, and FTIR analyses clearly confirm the successful exfoliation and formation of high-quality $Ti_3C_2T_x$ flakes with minimized defects.

Likewise, the HRTEM analysis of $Ti_3C_2T_x$ flakes confirmed the previously obtained data. For obtaining a high conductivity rate and boosted electromagnetic interferences (EMI) shielding, it is essential to maintain the size of $Ti_3C_2T_x$ flakes as large as possible while minimizing the generation of defects [15]. As depicted in Figures S2a-b, the $Ti_3C_2T_x$ flakes are perfectly exfoliated and present a well-defined 2D morphology with a large active surface area. The atomic morphology of the $Ti_3C_2T_x$ flakes (Figures S2c-d) further confirms this matter, indicating the formation of high-quality flakes with a well-arranged atomic morphology and the least vacancies. These outcomes confirm the successful synthesis of $Ti_3C_2T_x$ out of the primary MAX phase source with the desired quality, size, morphology, and surface functionalities.

GO flakes were also analyzed via diverse techniques to confirm their successful formation. Figure S3a shows the X-ray diffractogram of GO. As shown, GO presents a well-defined peak at 2θ of 11.15, corresponding to the (001) crystalline plane of GO and confirms the successful exfoliation of the graphite flakes [4, 16]. The Raman spectrum of GO shows two well-defined peaks at 1351.5 cm^{-1} and 1602.5 cm^{-1}, attributed to the D- and G-bands of GO flakes, respectively (Figure S3b) [3]. The ratio of I_D/I_G for the GO was measured to be 0.962, indicating the controlled rate of defects in the structure of exfoliated GO flakes. Figure S3c illustrates GO flakes' thermogravimetric analysis (TGA). As depicted, the GO showed a small weight loss at about 160 °C, corresponding to the water's volatilization. The major weight loss between 160 to 225 °C was caused owing to the decomposition of oxygen-based functional groups (-OH) along with the release of CO and CO_2. The other slight mass losses between 300 to 900 °C correspond to the detachment of more stable oxygen-based functional groups from GO flakes. The slight weight loss at more than 300 °C mainly originated from CO release [17].

The FTIR spectrum of GO also depicted the main fingerprint peaks of GO flakes, produced via the improved modified Hummers method (Figure S3c) [1]. Accordingly, the appeared peaks correspond to the stretching vibration of hydroxy functional groups (-OH) (3420 cm^{-1}), C=O in carbonyl moieties, and carboxylic acid groups (1740 cm^{-1}), C=C double bond carbon atoms (1640 cm^{-1}), C-O-C (1215 cm^{-1}), C-O (1090 cm^{-1}), and C-OH (1410 cm^{-1}) [17, 18]. The TEM images of the as-developed GO flakes (Figure S3e-f) depict the well-exfoliation of the GO and its 2D morphology. FESEM image of the GO (Figure S3g) also shows the successful exfoliation of graphite flakes and the formation of GO with a large active surface area, ideal for its subsequent decoration with magnetic nanoparticles, viz., Fe_3O_4.

Figure S4 illustrates the outcome of characterization for the decorated GO with Fe_3O_4 nanoparticles (mGO). Figure S4a shows the X-ray diffractogram of mGO; as depicted, the ultrasonic process and subsequent overnight stirring at 50 °C led to the reduction of the GO and shifted its 2θ peak from 11.15 to 18.50, indicating the decline in the d-spacing of the GO and reduction of GO flakes. Other appeared peaks in the X-ray diffractogram of mGO are generated owing to the decoration of Fe_3O_4 nanoparticles on the surface of GO. Correspondingly, 2θ peaks of 30.40, 35.60, 43.40, 53.60, 57.30, and 62.90 are attributed to the (220), (311), (400), (422), (511), (400) crystalline plane of magnetite nanoparticles; these outcomes are well matched with the standard card of JCPDS 19-0629, indicating the successful synthesis and high purity of magnetite nanoparticles [19]. The micro-Raman spectrum of the mGO (Figure S4b) shows the D-band and G-band peaks of GO at about 1347.5 and 1593.5 cm^{-1} with an I_D/I_G ratio of 0.886, indicating the formation of magnetic GO flakes with controlled defects. The outcome of Zeta potential for the mGO also showed that the as-developed compounds are negatively charged (-15 eV), leading to their stability and uniform distribution within the water [1].

The TGA graph of mGO reveals three main weight losses for the developed magnetic flakes (Figure S4c). In this regard, the first weight loss at about 120 °C could be attributed to water desorption, which includes 6% of the total weight of the compound. The second part starts from 150 °C up to 250 °C, corresponding to the degradation of oxygen-based functional groups. This weight loss is slightly continued till 750 °C, leading to the detachment of more stable functional groups from the surface of mGO. The third part is assigned to the degradation of GO flakes at high temperatures [20, 21].

Furthermore, the FESEM images of mGO indicate the homogeneous decoration of Fe_3O_4 nanoparticles on the surface of GO and the formation of 2D magnetic flakes (Figures S4d-e). Figures S4 f-g show the zoom area and the corresponding energy-dispersive X-ray spectroscopy (EDX) map of iron elements on the surface of GO, respectively, confirming their homogeneous distribution on the surface of GO. In this regard, the EDX analysis revealed that the mGO is comprised of 52.3 wt% iron, 31.0 wt% oxygen, 13.2 wt% carbon, 3.2 wt% nitrogen, and 0.3 wt% sulfur (Figure S4h).

3. Shielding setup and background concept

This section carefully evaluated the background concept of electromagnetic shielding and the techniques used to assess aerogel specimens. In this matter, the electromagnetic interference (EMI) shielding properties of the as-developed aerogels were examined within the X-band frequency range (8.2-12.4 GHz) using a vector network analyzer (VNA) (Keysight model P9374A) equipped with the WR-90 rectangular waveguide. The aerogel samples were attached to an offset and placed between the WR-90 waveguide adaptors connected to the VNA via microwave cables. Further information regarding the background concepts of EMI shielding and used formulations will be discussed in the following sections.

3.1. Conceptual mechanism of EMI shielding

Electromagnetic (EM) waves, known as synchronized oscillations of electric and magnetic fields, interact with a surface or interior of an EM shield through three different pathways: reflection, absorption, and multiple reflections. Owing to some morphological design and material selection of EM shields, another shielding phenomenon, namely multiple internal reflections or internal scattering, could occur that demonstrates the EMI shielding characteristic of an EM shield. The initial interactive mechanism between an EM wave and a shield is the reflection that occurs due to the impedance mismatch between the conductive shield or the conductive components within the shield and the free space [6, 22, 23, 24]. The impedance of an EM wave is known as the ratio of the transverse components of the electric and magnetic fields. In the conductive shields, the impedance becomes much lower compared to the free space (377 Ω) because of their conductive additives, leading to the inability of the incident EM waves to propagate within the conductive components. This process leads to the transfer of energy from the incident EM waves to the free-charged particles and electrons in the conductive shield, forcing them to oscillate. The enforced oscillating charges of the shield create an induced or scattered field known as "reflection." Importantly, the reflection mechanism mainly happens at the surface of an EM shield and chiefly depends on the electrical conductivity of the as-developed shielding structures [7, 24]. Along with the reflection mechanism, a portion of EM waves dissipates in the form of heat upon interacting with the EMI shielding materials/structures. This EM wave dissipating mechanism is known as "absorption" and occurs in a structure/material with finite electrical conductivity and electrical/magnetic dipoles [24, 25]; this mechanism is the ultimate goal of an EMI shielding system that should be achieved via smart structural design and component selection. Additionally, a portion of the incident EM waves also passes through the shielding structures/materials known as "transmission."

There are also other important mechanisms that could significantly affect the total shielding characteristic of an EMI shielding configuration, namely multiple reflections and internal scattering. In previous studies, these concepts were wrongly confused with each other [26, 27]. The multiple reflections mechanism occurs within thin EMI shielding films. In this regard, when incident EM waves pass through the front interface of a thin EMI shield, they might reflect back to the front interface upon interaction with the back interface, in which a portion of the reflected EM waves leaves the shield and the trapped ones re-reflects within the shielding structure to contribute within the second transmission. This process repeatedly happens upon the incident EM wave completely loses its energy and becomes totally dissipated. The multiple reflection process is chiefly dependent on the thickness of the shield and becomes negligible at thicknesses close to or larger than the skin depth, viz., a distance beneath the shield's front surface where the intensity of the electric field declines to 1/e of the incident wave's intensity. On the other hand, if the thickness of the thin film EM shield becomes smaller than the skin depth, multiple reflections between the front and back interfaces of the shield will decline the shielding effectiveness of the final structure [24, 28, 29].

Importantly, internal scattering or internal multiple reflections occurs within materials with numerous internal interfaces or designed porosities such as aerogels, foams, segregated structures, and multi-layered shields. Contrary to the multiple reflections, the internal scattering can promote the shielding performance of a shield and boost its shielding effectiveness by means of absorption via boosting the dissipation of infiltrated EM waves into the structure of shields with numerous interfaces or porosities. In this matter, the numerous interfaces within a porous or smartly designed configuration with mismatching impedances contribute to multiple back-and-forth reflections within the shield, prolonging the propagation pathway of EM waves prior to transmission, and

improving the effective interaction of the shield with EM waves till dissipating their energy. The numerous back-and-forth reflections between interfaces of a shield lead to a decline in the energy of the EM wave in the form of heat and increase the absorption rate of the shield, viz., contributing to the absorption mechanism.

Hence, the term internal scattering must be distinguished from the multiple reflections. The internal scattering refers to numerous internal interfaces, e.g., porosities, within the shield that traps the intruding EM waves and dissipates their energy in the form of heat via back-and-forth reflections between impedance mismatching interfaces. In contrast, multiple reflections refer to reflection between the front and back interfaces of a thin film shield that reduces the shielding effectiveness by letting the infiltrated EM waves escape the shield [24, 28, 30, 31]. Our work mainly focused on strategies to boost the internal porosities of worm-shaped aerogel filaments featuring well-arranged porosities by integrating GO, MXene, or magnetic GO nanosheets with each other. The employed strategy can boost the internal scattering of the shield by contributing to back-and-forth reflections between the impedance mismatching surfaces of MXene and GO or magnetic GO nanosheets, leading to tremendous dissipation of infiltrated or trapped EM waves within the structure of aerogels. Additionally, the design of the Janus aerogel also provides the possibility to reach a 3D-designed hybrid layered structure in which the non-conductive magnetic or non-magnetic and conductive layers containing MXene nanosheets were assembled along each other, leading to a remarkable shielding performance by record high absorption-based mechanism. In the next section, the formulations and EMI shielding parameters will be discussed in detail.

3.2. EMI shielding parameters and formulations

In this study, a two-port VNA is employed to characterize the shielding performance of the as-developed aerogels. In this approach, the system sends a signal from port one (S_1) with a single

defined frequency to the sample. The receiver detects the transmitted (S_{21}) and reflected (S_{11}) waves and subsequently measures the magnitude and the phase data of each received signal. The same operation occurs from port two (S_2), and the devised system sends the same signal at the same frequency to the sample, measuring the transmitted (S_{12}) and reflected (S_{22}) waves by the receiver on the opposite side. When the device shifts to the next frequency, the measurements occur again. At the end of the assessment, the transmission and reflection measurements are provided in terms of complex scattering, i.e., S- parameters. Importantly, the homogeneity of the sample will result in equal values of S_{11} and S_{22}, as well as S_{21} and S_{12} [8].

The obtained scattering parameters are employed to measure the EMI shielding coefficients, including reflectance (R), absorbance (A), and transmittance (T). The responsive EMI shielding mechanism of the developed aerogels can be precisely assessed according to the obtained EMI shielding coefficients. The R coefficient (reflection) is defined as the power of the reflected wave (P_R) divided by the incident wave (P_I). This coefficient can be calculated from the complex S_{11} parameter via the following formula:

$$R = \frac{P_R}{P_I} = S_{11}^{\ 2} \quad (1)$$

in which the S_{11} is the complex S- parameter calculated according to the reflected voltage magnitude divided by the incident voltage magnitude in port S_1. Additionally, the T coefficient (transmittance) is known as the power of the transmitted wave (P_T) divided by the incident wave that can be calculated via the complex S_{21} parameter:

$$T = \frac{P_T}{P_I} = S_{21}^{\ 2} \quad (2)$$

where S_{21} is the transmitted voltage magnitude from port 1 to port 2 divided by the incident voltage magnitude in port 1. The A coefficient (absorbance), which is the dominant goal of an effective shield, can be measured via the following equation:

$$A = 1 - R - T \quad (3)$$

3.3. Shielding effectiveness and logarithmic parameters

The shielding effectiveness, known as the EMI shielding effectiveness (SE_T), is another essential parameter that is widely used for judging the total shielding performance of an EMI shield. The SE_T parameter is the safeguarding performance of a shield against incident EM waves, defined as the total power of transmitted EM waves divided by the total power of the incident EM waves on a logarithmic scale, which can be calculated via the following equation:

$$SE_T(dB) = 10\log\frac{P_T}{P_I} = 20\log\frac{E_T}{E_I} \quad (4)$$

in which the P and E parameters correspond to the power intensity and intensity of the electric field, respectively. The subscripts T and I are attributed to the transmitted and incident EM waves, respectively. According to the Schelkunoff's theory, the EMI shielding effectiveness, i.e., SE_T, is defined as the sum of shielding performance resulting from the reflection loss (SE_R), absorption loss (SE_A), and multiple reflections loss (SE_M) [32, 33, 34, 35]:

$$SE_T = SE_R + SE_A + SE_M \quad (5)$$

The magnitude of the reflection loss or SE_R for both front and back interfaces of the shield can be precisely measured by using the simplified version of Fresnel's equation for a monolithic conductive shield that could be defined as following [28]:

$$SE_R(dB) = 20\log\frac{(\eta+\eta_0)^2}{4\eta\eta_0} = 39.5 + 10\log\frac{\sigma}{2\pi f\mu} = 10\log\left(\frac{1}{1-R}\right) \quad (6)$$

in which parameters η and η_0 are attributed to the impedance of the shield and air in Ω, respectively. Other parameters such as μ, σ, and f correspond to the magnetic permeability, electrical conductivity, and the incident EM wave's frequency in Hz, respectively. As evidenced

in equation 6, the SE_R has a direct relationship with the electrical conductivity, and upon an increase in the shield's electrical conductivity, the reflection loss will increase accordingly. Likewise, the magnetic permeability and the frequency of the incident EM waves also play a crucial role in the total magnitude of the reflection loss [28].

Upon propagation of an EM wave into a lossy medium with finite electrical conductivity and electrical/magnetic dipoles, the infiltrated EM wave will be attenuated upon energy dissipation. The dissipated energy will be transformed into the form of heat that can be expressed as the attenuation constant (α) and be calculated via the following formulation [29, 36]:

$$\alpha = \sqrt{\frac{\mu\varepsilon}{2}\left[\sqrt{1+\left(\frac{\sigma}{\omega\varepsilon}\right)^2}-1\right]} \quad (7)$$

where ε and ω correspond to the dielectric permittivity and angular frequency (2πf). When an EM wave enters a lossy medium, the strength or amplitude (E) of the EM wave abates exponentially to $E = E_0 e^{-\alpha} d$ within an EMI shield with a thickness of d. Effective attenuation of the incident EM waves has several requirements, such as I) the finite electrical conductivity for effectual ohmic losses that contribute to the interaction of available electrons in the shield with the incident EM wave [37], high magnetic permeability to improve the magnetic losses that are accompanied with the magnetic hysteresis loss and eddy current loss [38], and high dielectric permittivity for efficacious dielectric losses that occurs due to the generation of multiple nano and/or micro capacitors [39]. The total absorption loss (SE_A) of the shield can be measured via the following equation:

$$SE_A(dB) = 20\log e^{\alpha d} = 20\left(\frac{d}{\delta}\right)\log_{10} e = 8.68\left(\frac{d}{\delta}\right) = 8.7d\sqrt{\pi f \mu \sigma} = 10\log\left(\frac{1-R}{T}\right) \quad (8)$$

in which δ is attributed to the skin depth or EM wave's penetration depth in μm. The skin depth is a highly important parameter in the shielding measurement because it expresses the distance

beneath the shield's top interface in which the electric field's intensity declines to 1/e of the initial intensity of the incident EM wave. For an electrically conductive shield, the skin depth can be calculated via the following formula:

$$\delta = \frac{1}{\alpha} = \left(\sqrt{\pi f \sigma \mu}\right)^{-1} \quad (9)$$

According to the formulation of the absorption loss (equation 8), a soar in the electrical conductivity and thickness of a shield can improve the absorption loss, whereas permittivity and permeability are employed to measure the absorption loss. Additionally, in a thin film shield or a shield with a small thickness, the reflection from the bottom interface of the shield affects the final transmission owing to the multiple reflections, where the reflected EM wave from the bottom interface re-reflect after interaction with the top interface and lead to a second transmission. Continuing in this process causes multiple reflections between a shield's top and bottom interfaces, leading to a lower value of SE_T for the shield. The multiple reflection loss (SE_M) can be measured via the following equation:

$$SE_M(dB) = 20\log_{10}\left(1 - e^{-\alpha d}\right) = 20\log_{10}\left[1 - e^{-\frac{2d}{\delta}}\right] \quad (10)$$

Another important parameter for lightweight shields, especially aerogels, is the specific shielding effectiveness (SSE) (dB cm^2 g^{-1}) that considers both a shield's density and thickness. This parameter can be measured via the following equation [37]:

$$SSE = \frac{SE_T}{\rho * t} \quad (11)$$

in which the ρ is the density (g cm^{-3}), t is the thickness (cm), and SE_T is the EMI shielding effectiveness (dB). This parameter shows the shield's effectiveness for the application that the weight is a crucial parameter such as lightweight shields based on the aerogel systems.

3.4. Shielding mechanism evaluation criteria and common mistakes

The dominant shielding mechanism of an EMI shielding structure must be assessed via shielding coefficients, i.e., R and A values. In fact, by considering the ratio of the incident EM waves' power to the transmitted and reflected powers of EM waves, it is possible to accurately determine if the shielding mechanism of an EMI shield is based on either absorption or reflection mechanism. Correspondingly, if the magnitude of the R coefficient becomes higher than the A coefficient, the dominant mechanism of the shield is based on the reflection mechanism. This matter happens for highly conductive shields owing to the impendence mismatch between the free space and the conductive shield, which leads to the generation of secondary reflections in response to the incident EM waves. In contrast, a higher A value than R demonstrates the dominant absorption mechanism, viz., $A>0.5$: absorption mechanism and $A<0.5$ or $R>0.5$: reflection mechanism, showing a higher rate of EM waves dissipation and their subsequent transformation to heat [22]. However, some recent studies mistakenly used the logarithmic values of SE_A and SE_R to judge the shielding mechanism of EMI shielding structures [26, 27]. Based on their logic, if $SE_A>SE_R$ or $SE_A/SE_R>1$, the dominant shielding mechanism is absorption. This statement is entirely wrong, as the shielding mechanism must be assessed based on the shielding coefficients as indicators of the total power of EM waves rather than the SE parameters that illuminate the logarithmic ratios of power for EM waves [22].

As a clarification, by considering the equation of the absorption loss ($SE_A=10\log\left(\frac{1-R}{T}\right)$), it can be seen that this parameter is defined as the ratio of the penetrated waves to the shield (after deduction of the reflectance from the incident waves) over the transmitted waves; hence, it is not the indicator of the ratio of the incident EM waves power over the reflected or transmitted EM waves power. Thereby, the shielding mechanism must be judged based on the shielding coefficient instead of the logarithmic values of SE parameters.

For instance, in a developed shield by Ghaffarkhah et al. [8], they reported shielding A, R, and T coefficients of 8.54×10^{-3}, 0.9914561, and 2.35×10^{-7}, respectively. Accordingly, to the obtained outcomes, this shield reflects about 99.14 % of incident EM waves (R>0.5) and only absorb or dissipate about 0.85 % of the EM waves. Hence, the dominant shielding mechanism of this shield is based on the reflection mechanism. On the contrary, they reported SE_A and SE_R values of 47.095 and 21.043 (much higher SE_A than SE_R or SE_A/SE_R=2.238>1). Herein, if the mechanism is judged based on SE parameters, it becomes absorption dominant, while the shielding coefficients demonstrate an opposite outcome. Therefore, the shielding coefficient must be used instead of logarithmic SE parameters to judge the shielding mechanism of an EMI shielding structure.

4. Figures

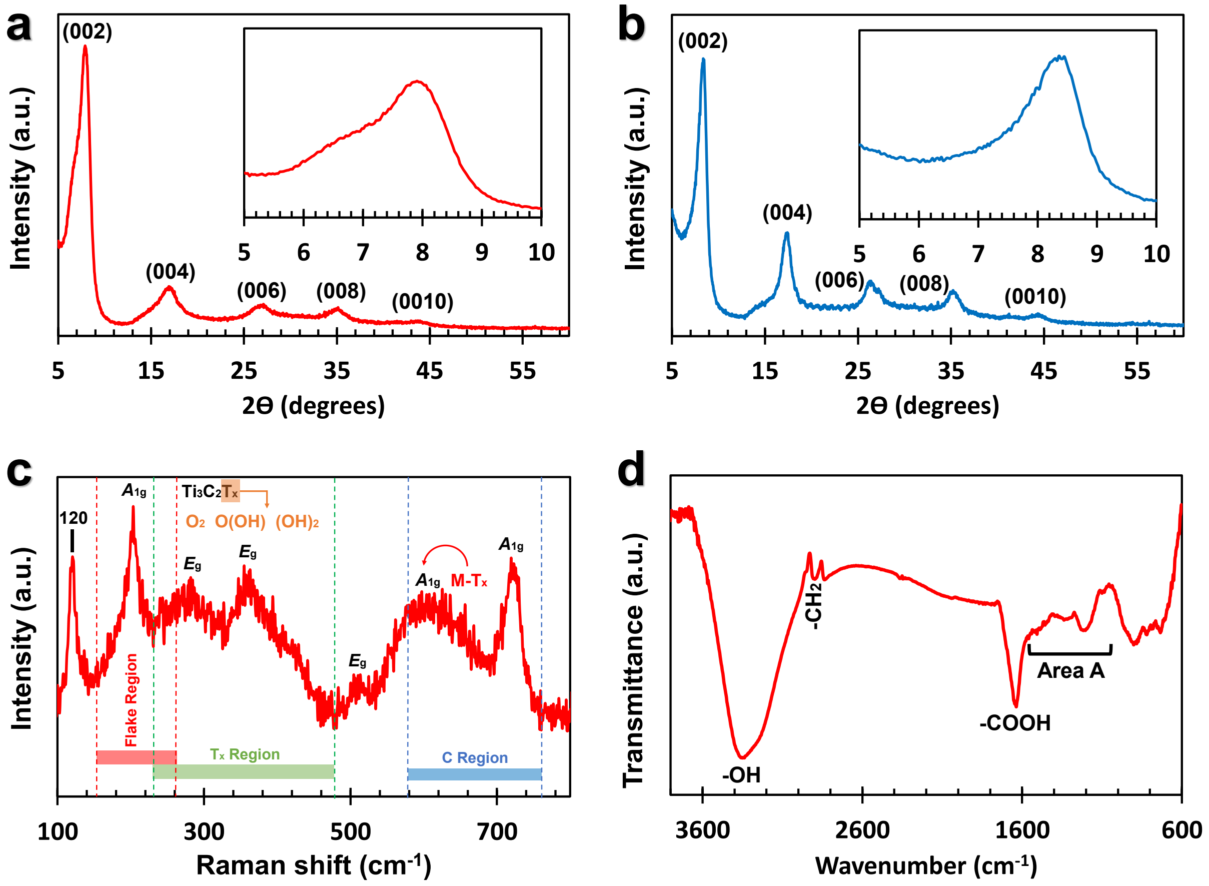

Figure S1: X-ray diffractogram of $Ti_3C_2T_x$ laminated films that annealed at (a) 60 °C and (b) 250 °C. (c) Micro-Raman spectroscopy and (d) FTIR analyses of $Ti_3C_2T_x$. (a-d) Reproduced based on our previous open-access article released under the Creative Commons Attribution-NonCommercial License, which allows for the reproduction and reuse of the data upon proper citation and attribution.[40]

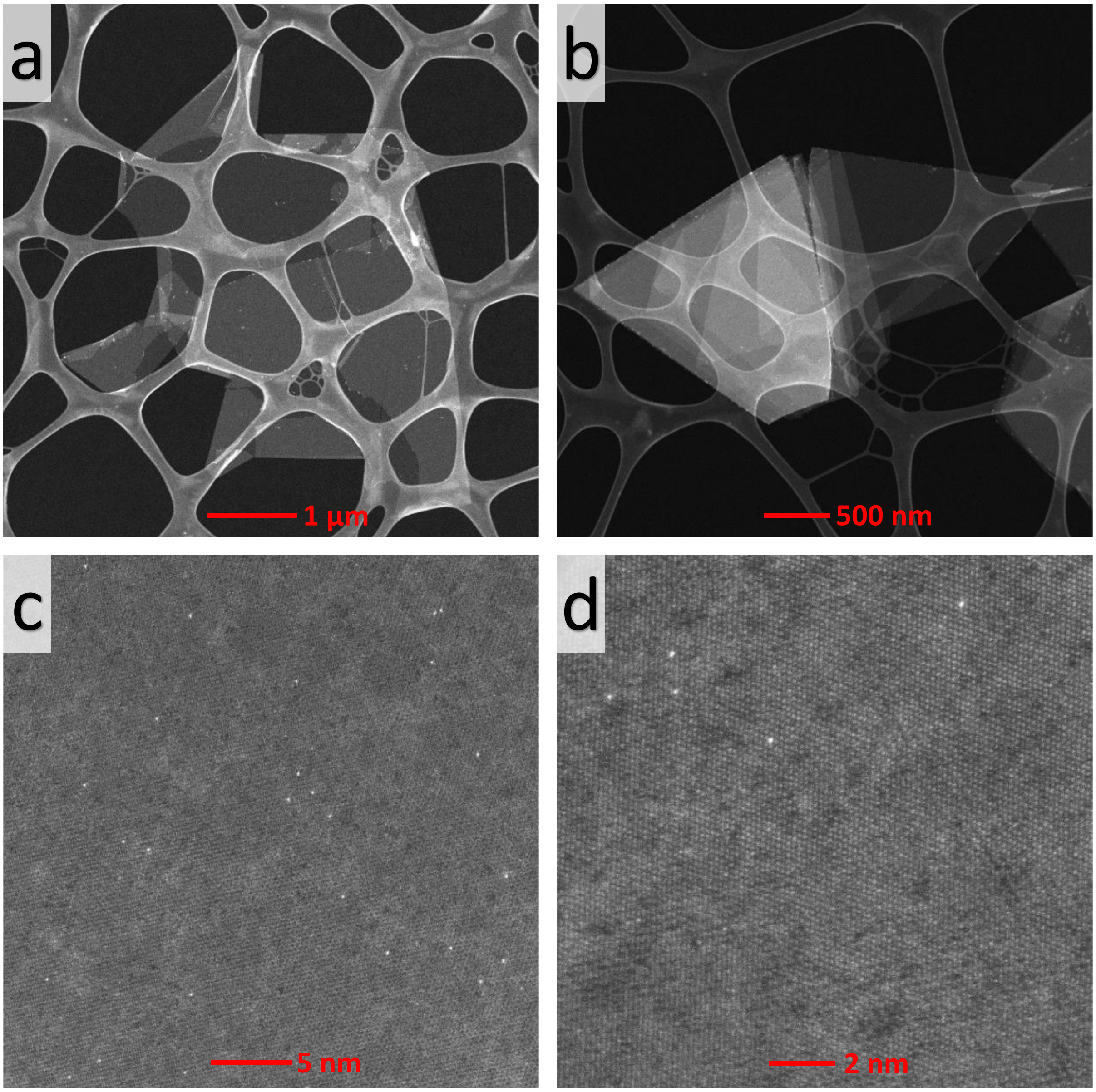

Figure S2: HRTEM analysis of $Ti_3C_2T_x$ flakes.

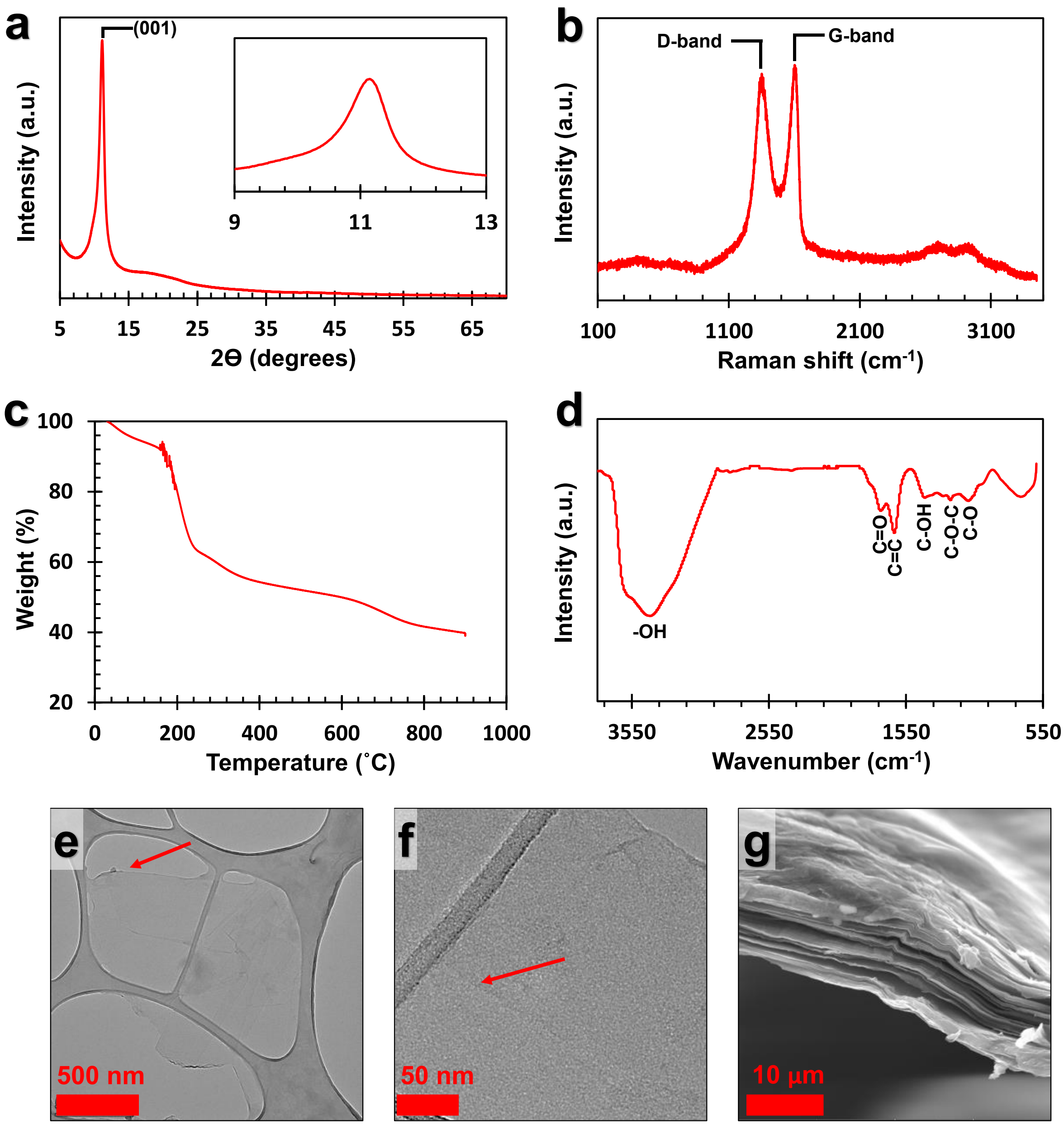

Figure S3: (a) X-ray diffractogram, (b) Micro-Raman spectra, (c) TGA, (d) FTIR spectra, and (e-f) TEM images of GO. (f) FESEM images of the cross-section of a GO film after vacuum filtration. (a-d) Reproduced based on our previous open-access article released under the Creative Commons Attribution-NonCommercial License, which allows for the reproduction and reuse of the data upon proper citation and attribution.[40]

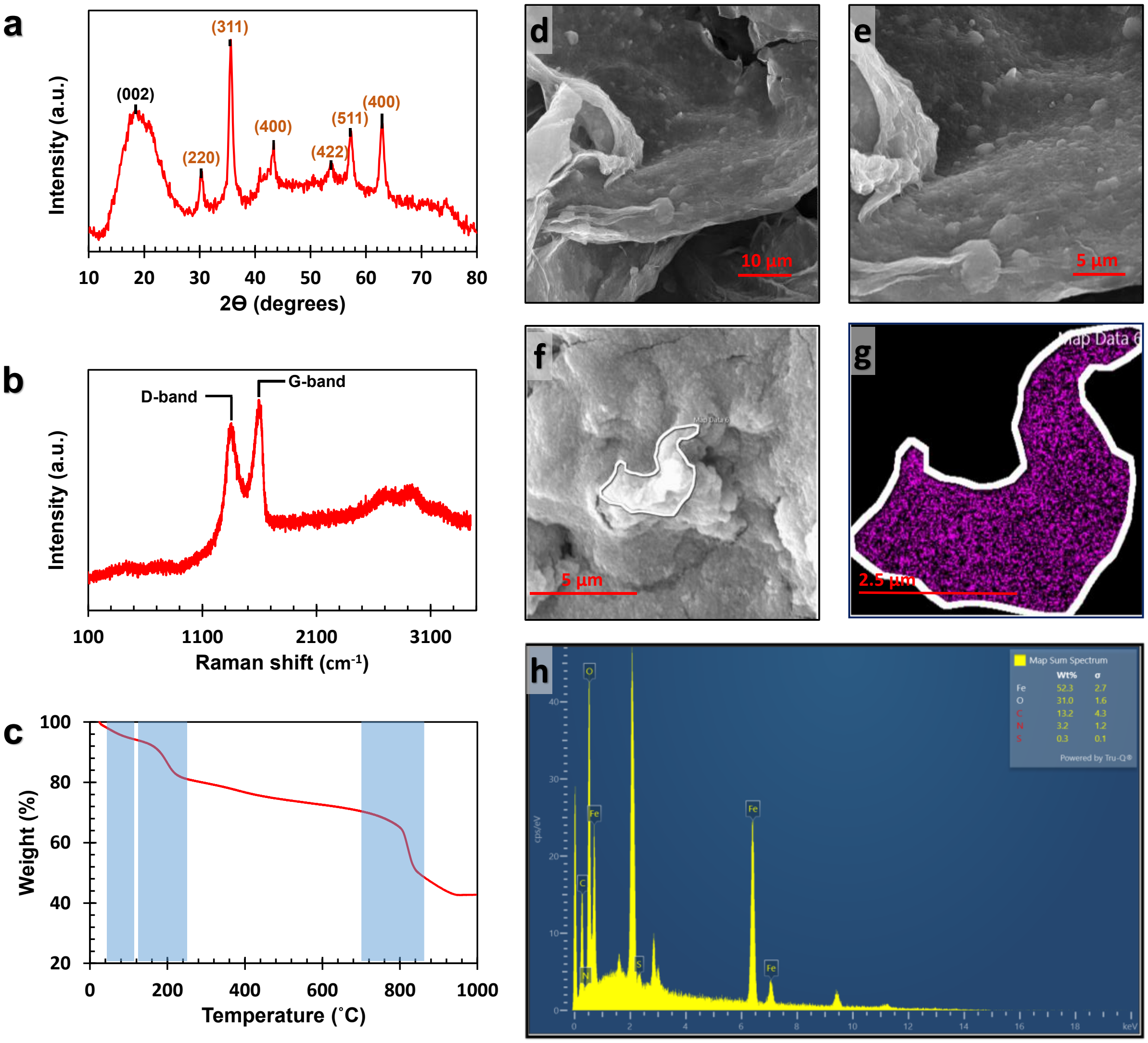

Figure S4: (a) X-ray diffractogram, (b) Micro-Raman spectra, (c) TGA, (d-e) FESEM, and (f-g) EDX analysis of mGO. The EDX map presented in part (d) has been taken from the section completely covered by Fe_3O_4.

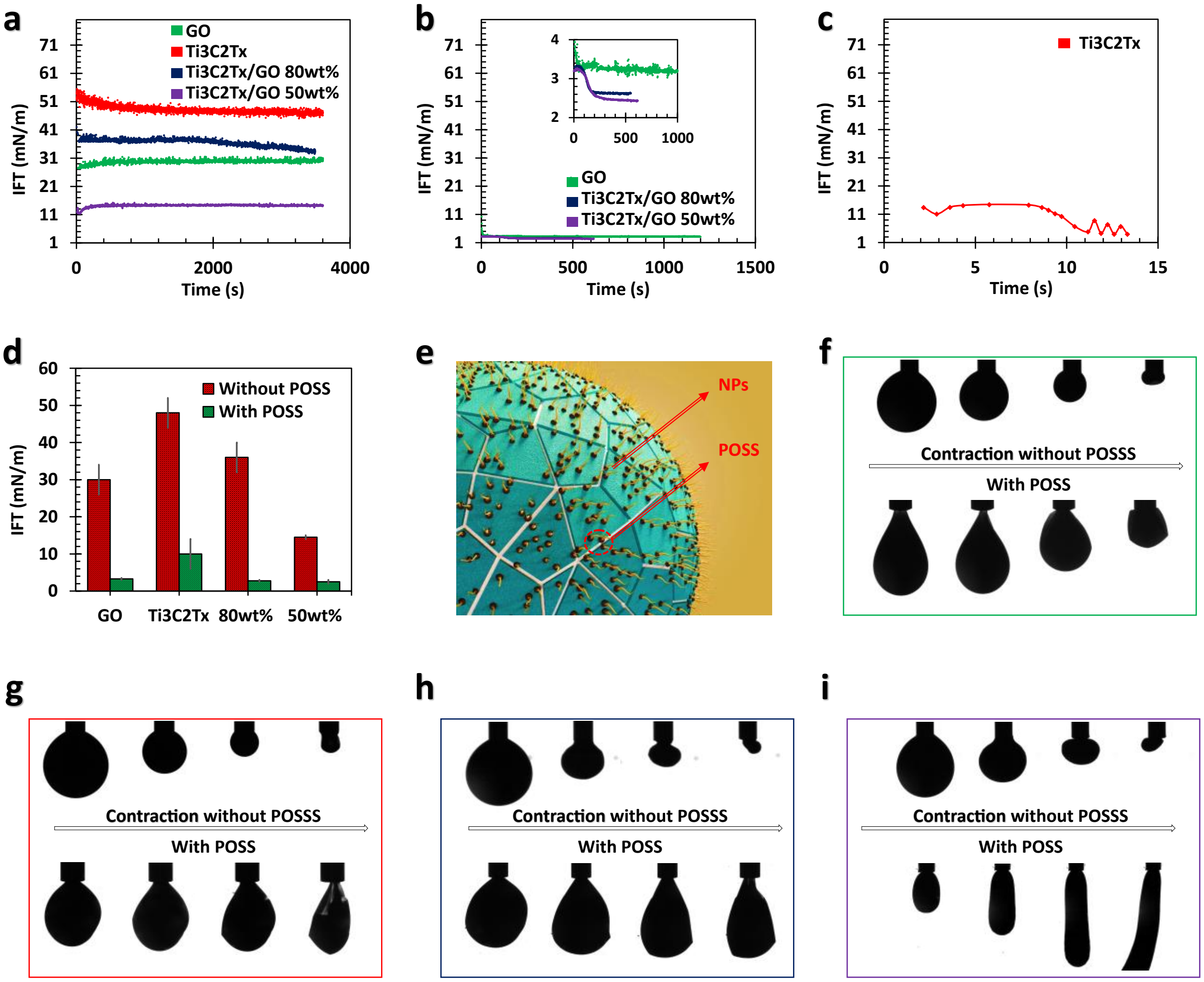

Figure S5: Interfacial characterization of NPs aqueous suspension in hexane domain (a) without and (b, c) with POSS. The aqueous suspension of $Ti_3C_2T_x$ (10 mg/ml), GO (10 mg/ml), and hybrid systems containing 20 and 50 wt% GO, i.e., 2 and 5 mg/ml of GO in 10 mg/ml $Ti_3C_2T_x$/GO suspensions, were tested in this experiment. (d) The average equilibrium IFT of the aqueous suspensions/hexane with and without POSS. (e) The schematic illustration of NPSs assemblies in the presence of POSS results in lower equilibrium IFT of the aqueous suspensions/hexane. Contraction of a pendent drop of (f) GO (10 mg/ml); (g) $Ti_3C_2T_x$ (10 mg/ml); (h) $Ti_3C_2T_x$/GO 80 wt%; and (i) $Ti_3C_2T_x$/GO 50 wt% in hexane with and without 1 mg/mL POSS. All error bars in (d) represent the standard deviation.

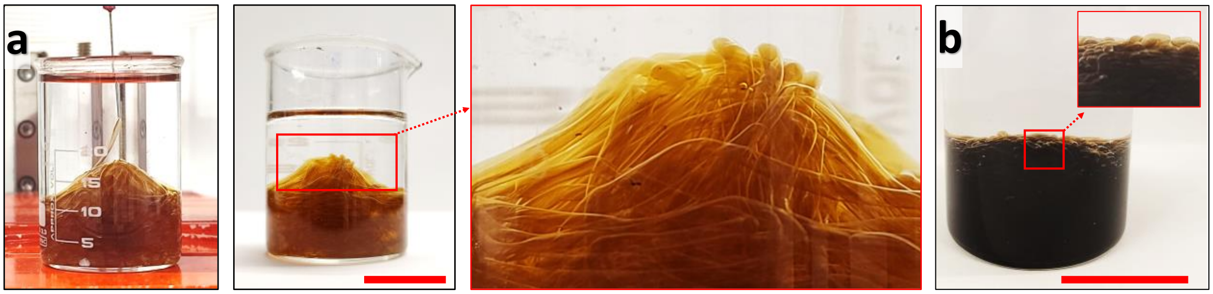

Figure S6: (a) Digital image demonstrating tubule formation upon streaming GO aqueous suspension (5 mg/ml) into hexane-POSS. (b) GO-liquid threads with a worm-like morphology after three months of aging. All scale bars in (a-b) correspond to 2 cm.

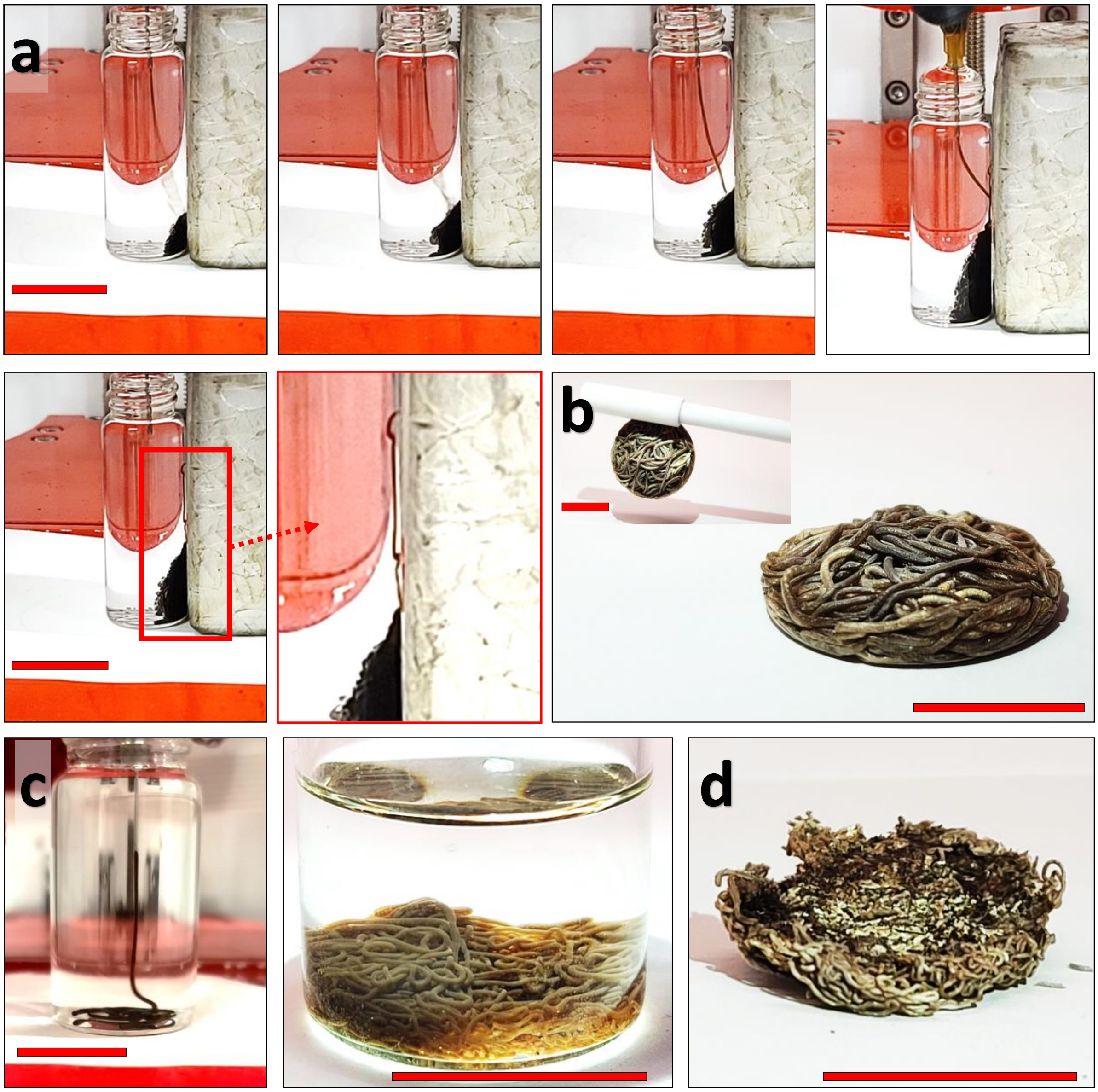

Figure S7: (a) The process of streaming mGO/GO 50 wt% and their response to an external magnetic field. (b) The prepared mGO/GO showed enhanced structural integrity and minimum shrinkage upon freeze-drying. (c) Digital image demonstrating tubule formation upon streaming 10 mg/ml mGO aqueous suspension. The mGO aqueous suspension is capable of fabricating liquid threads. (d) However, upon freeze-drying, relatively low structural stability and high-volume shrinkage for mGO aerogels were observed. All scale bars in (a-d) correspond to 2 cm.

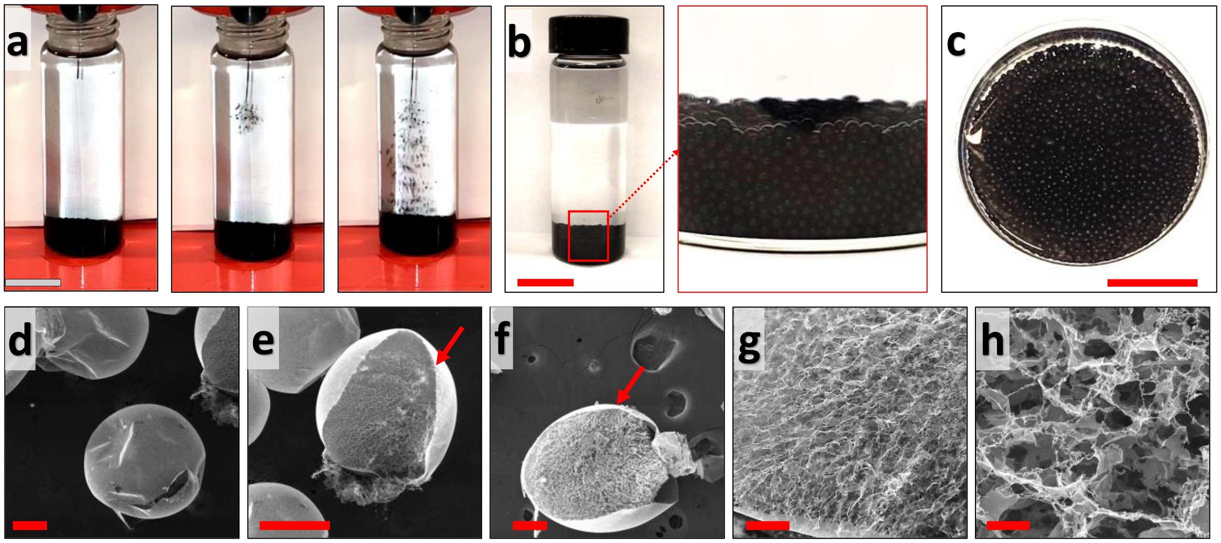

Figure S8: (a) Digital image demonstrating bead formation upon streaming $Ti_3C_2T_x$ aqueous suspension (10 mg/ml) into hexane-POSS. $Ti_3C_2T_x$ beads (b) before and (c) after three months of aging. (d-h) FESEM images of $Ti_3C_2T_x$ aerogel beads. These structures showcase laminated skin covering the bulk of aerogels. Scale bars in (a-c), (e), (d, f), (g), and (h) corresponded to 2cm, 400, 200, 50, and 10 µm, respectively.

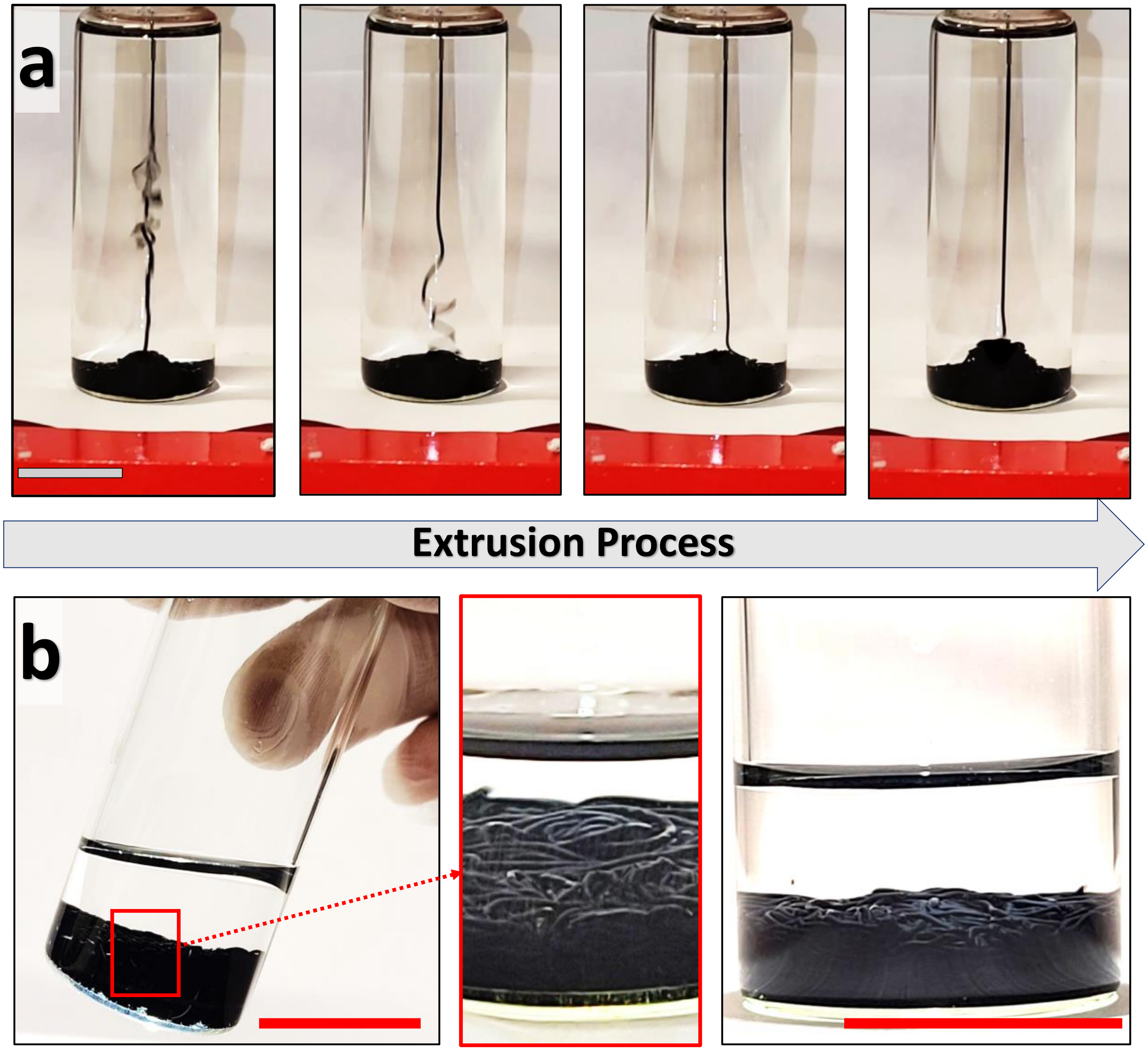

Figure S9: (a) Digital image demonstrating tubule formation upon streaming 10 mg/ml $Ti_3C_2T_x$ /GO aqueous containing 50 wt% GO. (b) $Ti_3C_2T_x$/GO-liquid threads with a worm-like morphology. The inner diameter of the nozzle and extrusion pressure were 330 μm and 10 psi, respectively. All scale bars in (a-b) correspond to 2 cm.

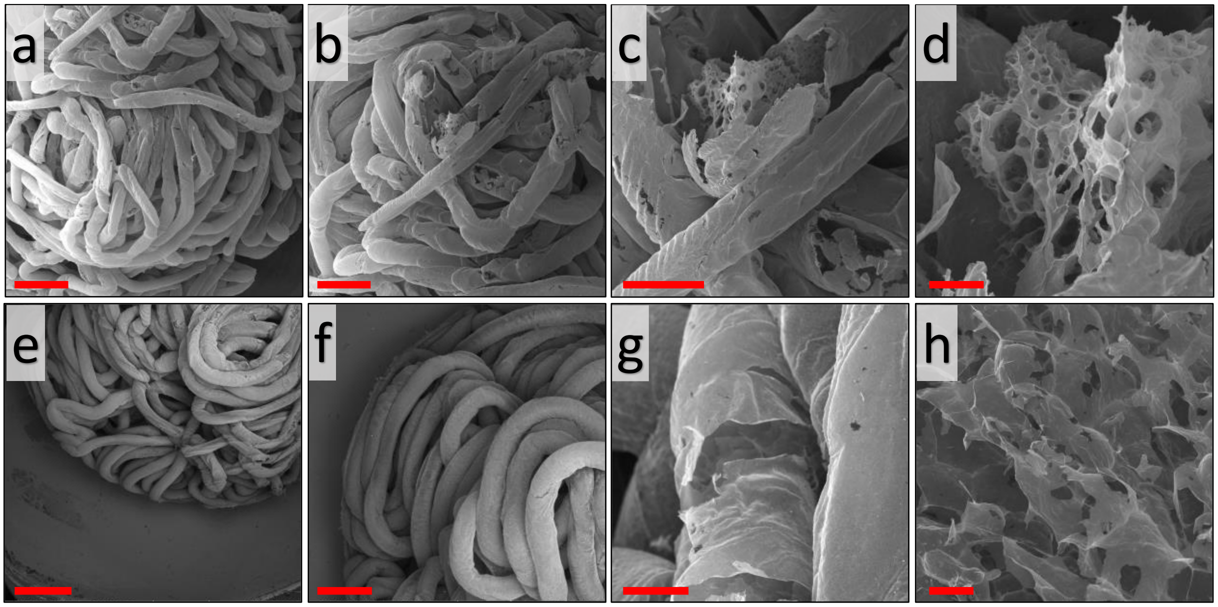

Figure S10: The FESEM images of worm-like aerogels prepared by $Ti_3C_2T_x$ /GO (a-d) 80 wt%- 10 mg/ml and (e-h) 50 wt%- 10 mg/ml inks. Both structures showcase laminated skin covering the bulk of these aerogels. Scale bars in (e), (a-b, f), (c), (g), (d), and (h) corresponded to 2000, 1000, 500, 200, 100, and 20 µm, respectively.

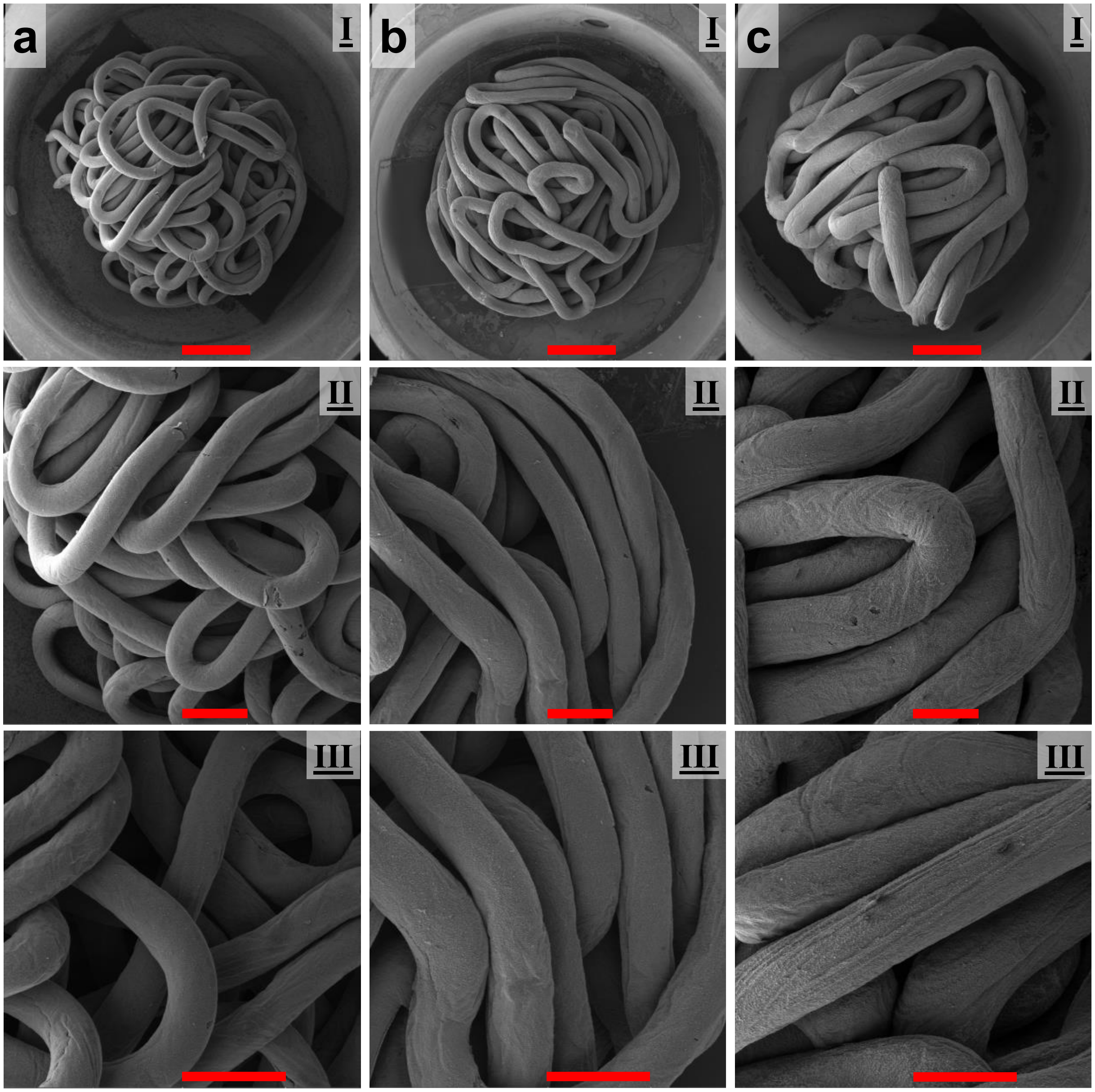

Figure S11: FESEM images of bulk and filaments of $Ti_3C_2T_x$/GO (20 wt% GO) aerogels generated via liquid streaming approach using needles with different gauge numbers of (a) 21, (b) 18, and (c) 15. In the case of these samples, the extrusion pressure was fixed at 15 psi. Scale bars equal (I) 2 mm and (II-III) 1 mm.

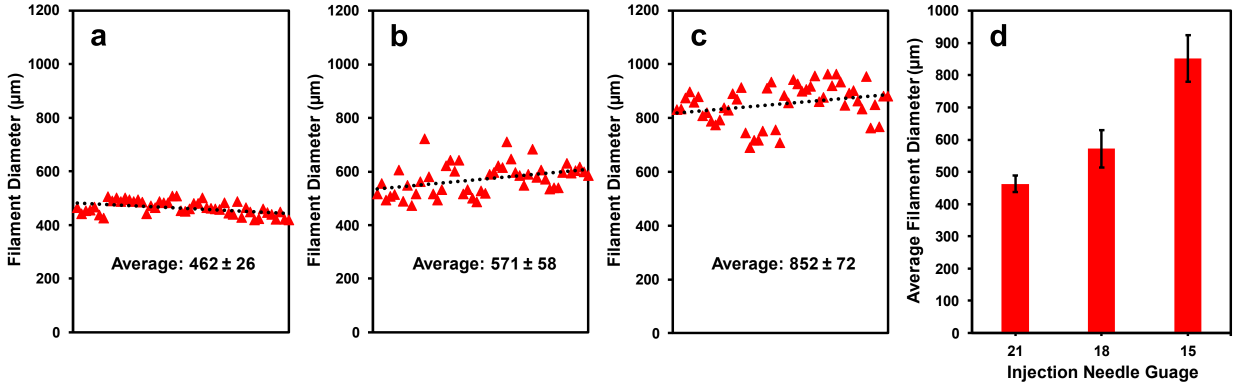

Figure S12: Filament diameter of $Ti_3C_2T_x$/GO (20 wt% GO) aerogels generated via liquid streaming approach using needles with different gauge numbers of (a) 21, (b) 18, and (c) 15. Average filament diameter of $Ti_3C_2T_x$/GO (20 wt% GO) aerogels prepared at different needle gauges. In the case of these samples, the extrusion pressure was fixed at 15 psi. All error bars represent the standard deviation.

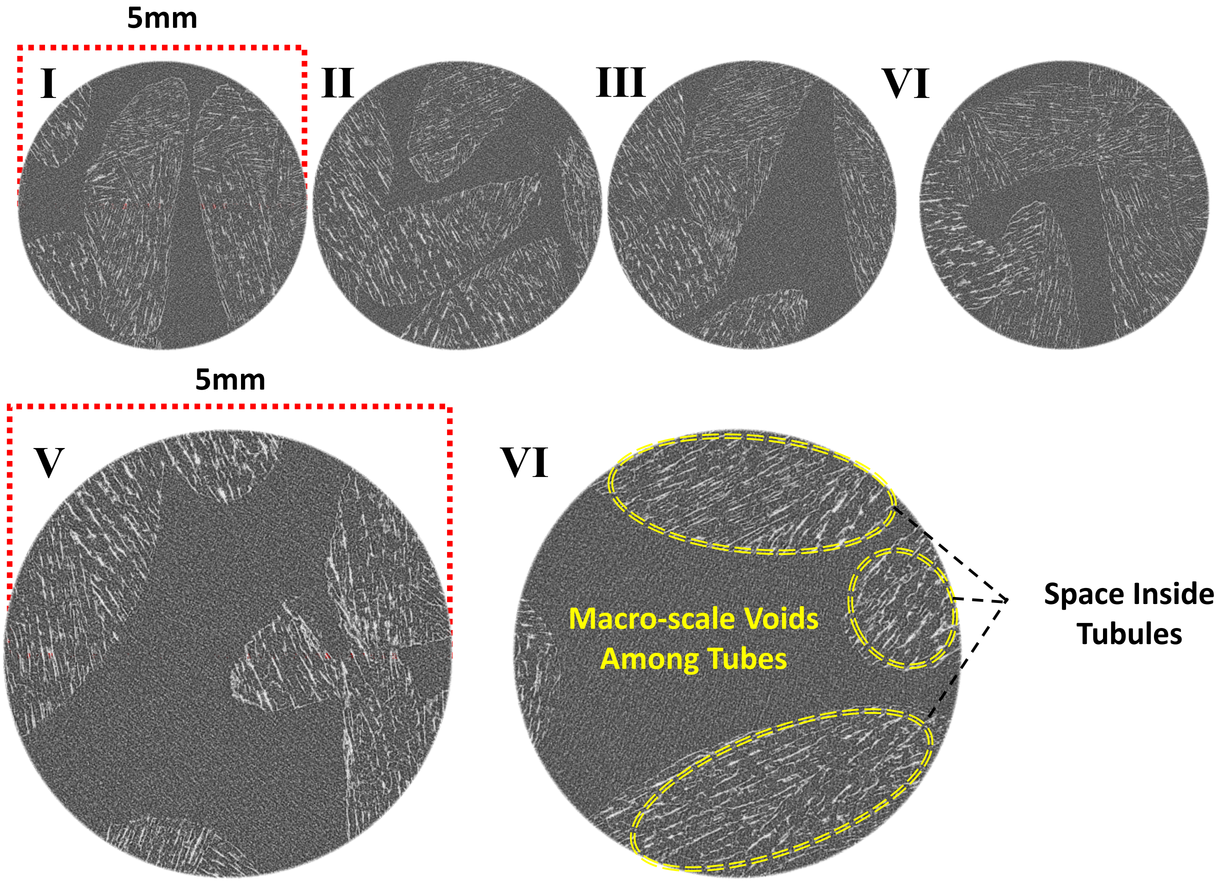

Figure S13: Cross-sectional micro-CT images of $Ti_3C_2T_x$/GO (20 wt% GO) aerogels prepared via gauge 15 needles. In the case of these samples, the extrusion pressure was fixed at 15 psi.

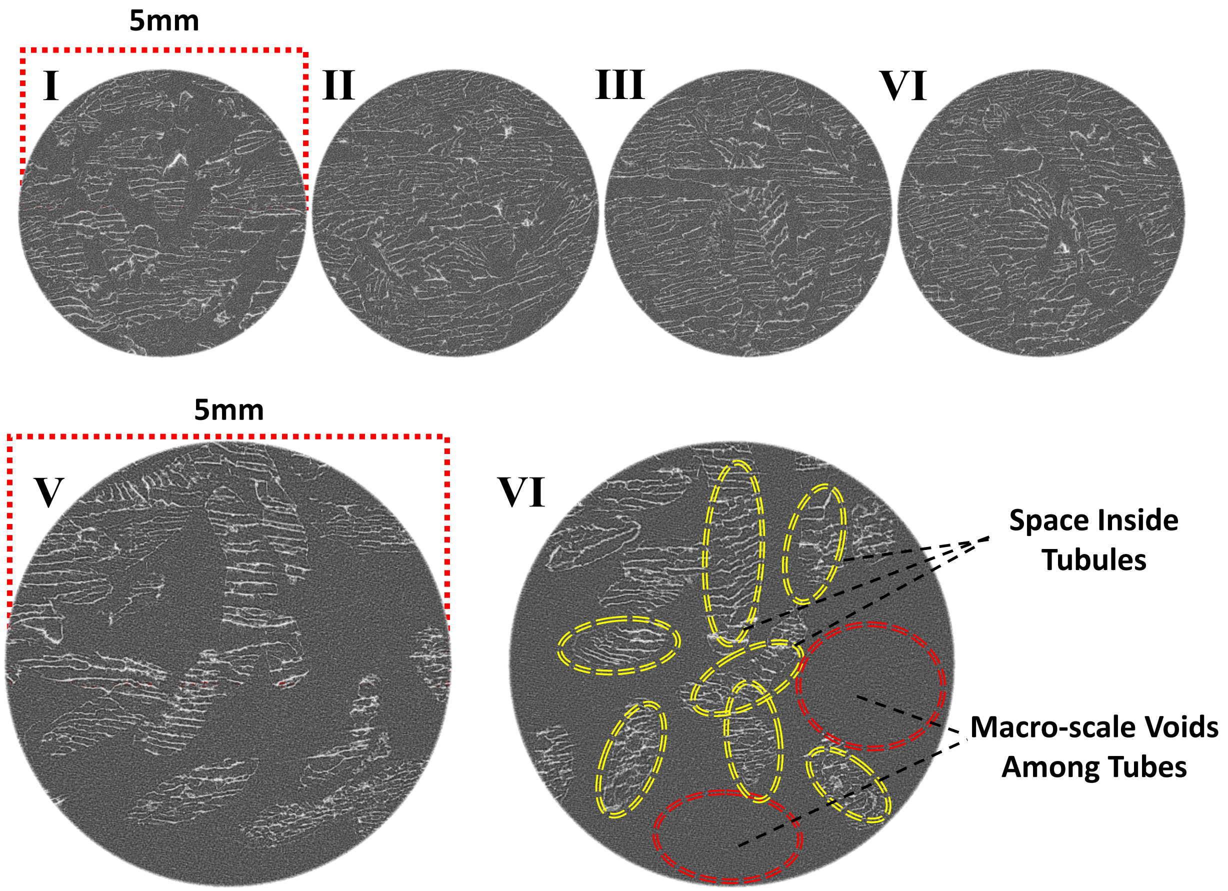

Figure S14: Cross-sectional micro-CT images of $Ti_3C_2T_x$/GO (20 wt% GO) aerogels prepared via gauge 21 needles. In the case of these samples, the extrusion pressure was fixed at 15 psi. It's worth emphasizing that, when employing smaller needles for extruding filaments, the section of the sample situated nearer to the bottom of the holding vials demonstrates a denser composition with diminished macro-scale voids, as evident in images I to VI.

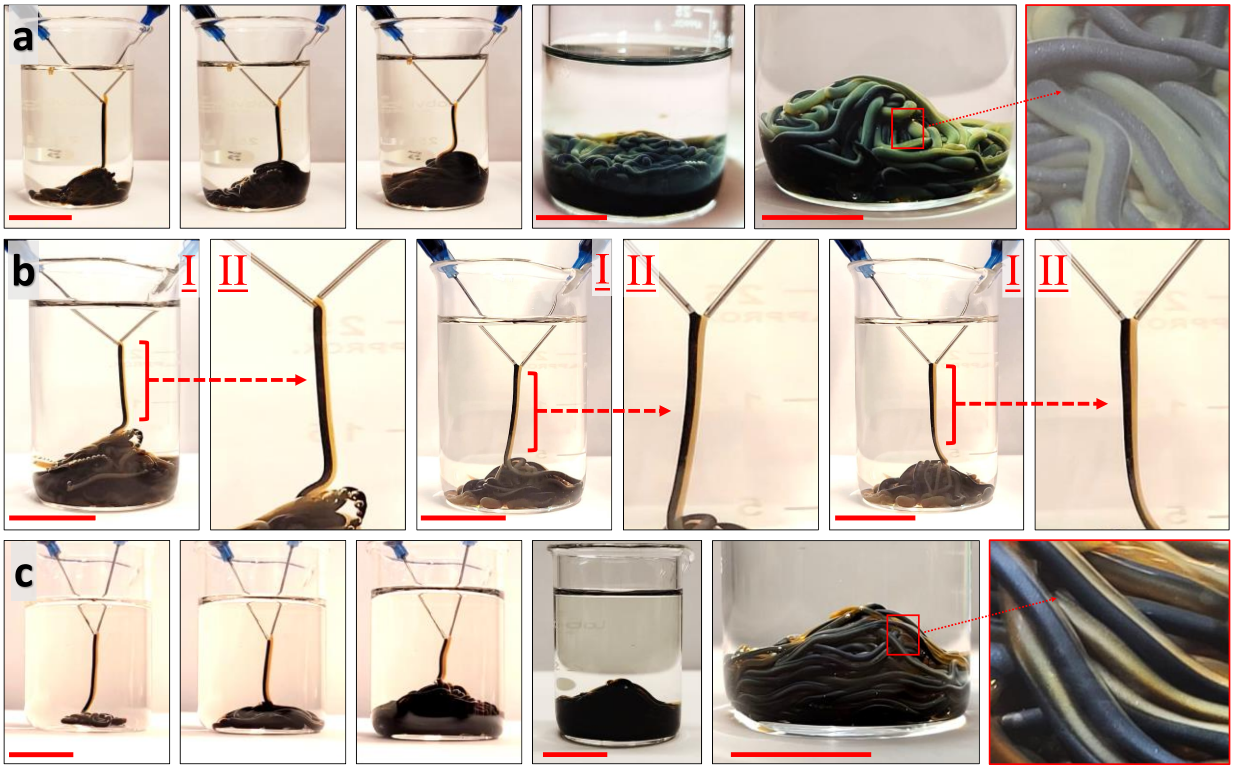

Figure S15: Digital image demonstrating Janus liquid threads formation. (a-b) Janus structures were prepared by merging streams of $Ti_3C_2T_x$/GO-10 mg/ml (20 wt% GO) and mGO/GO-10 mg/ml (50 wt% GO) inks. (c) Janus structures were prepared by merging streams of $Ti_3C_2T_x$/GO-10 mg/ml (20 wt% GO) and pure GO-10 mg/ml inks. All scale bars in (a-c) correspond to 2 cm.

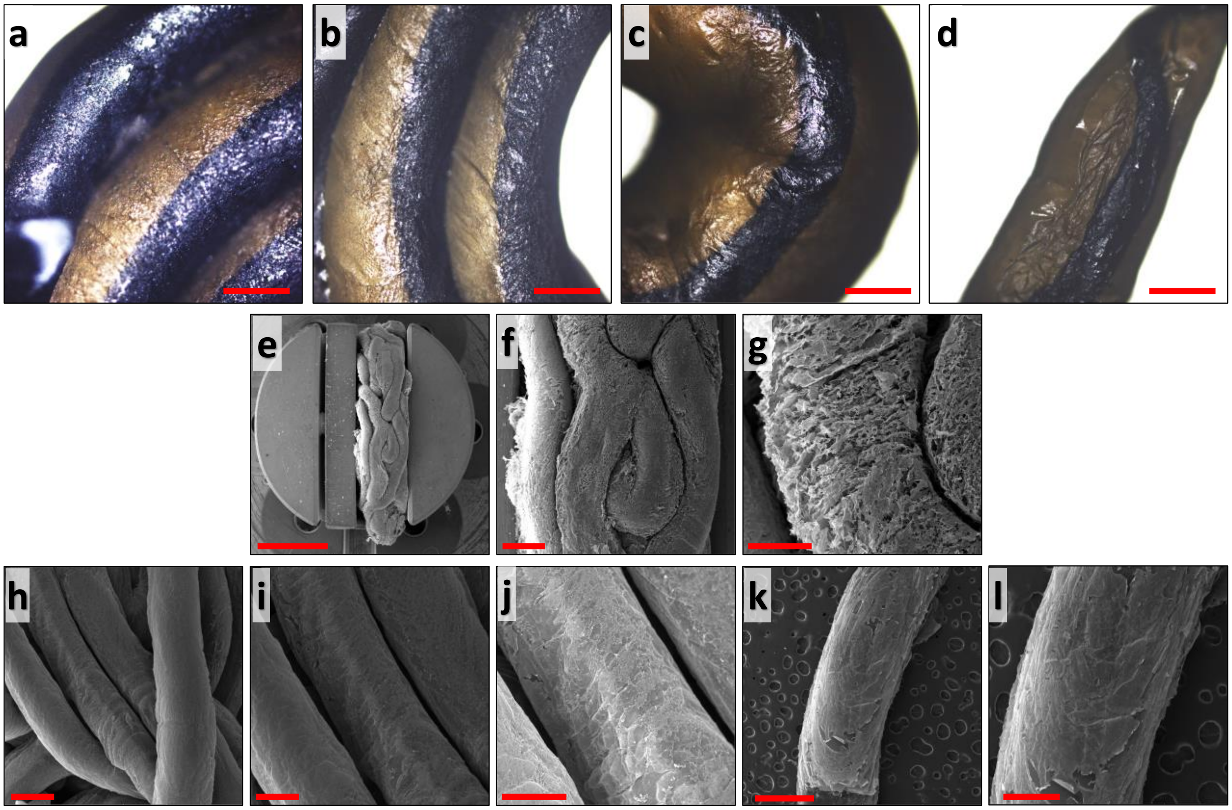

Figure S16: (a-d) Confocal images of the Janus liquid threads prepared by merging streams of $Ti_3C_2T_x$/GO-10 mg/ml (20 wt% GO) and mGO/GO-10 mg/ml (50 wt% GO) inks. In Figures a-b, 200 µm nozzles were used for both inks. However, in Figures c-d, we used different needles for each ink, i.e., 200 µm for $Ti_3C_2T_x$/GO and 400 µm for mGO/GO, and fabricated Janus structures in which the magnetic parts' volume is larger than that containing $Ti_3C_2T_x$. The FESEM images of (e-g) $Ti_3C_2T_x$/GO:mGO/GO and (h-l) $Ti_3C_2T_x$ /GO:GO aerogels. Scale bars in (e), (f, h, k), and (a-d, g, i-j, l) corresponded to 2000, 1000, and 500 µm, respectively.

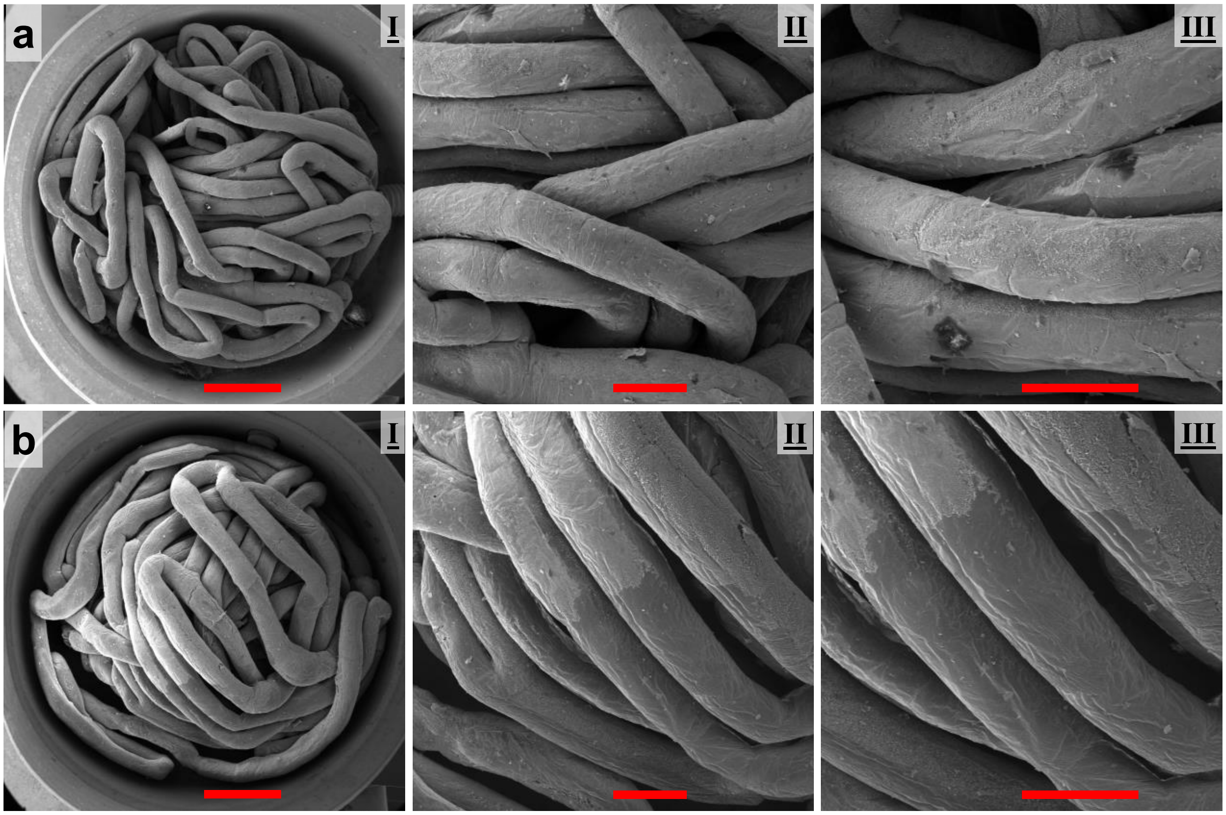

Figure S17: FESEM images of $Ti_3C_2T_x$/GO (20 wt% GO):GO Janus aerogels generated using needles with different gauge numbers of (a) 21 and (b) 18. Scale bars correspond to (I) 2 mm and (II-III) 1 mm.

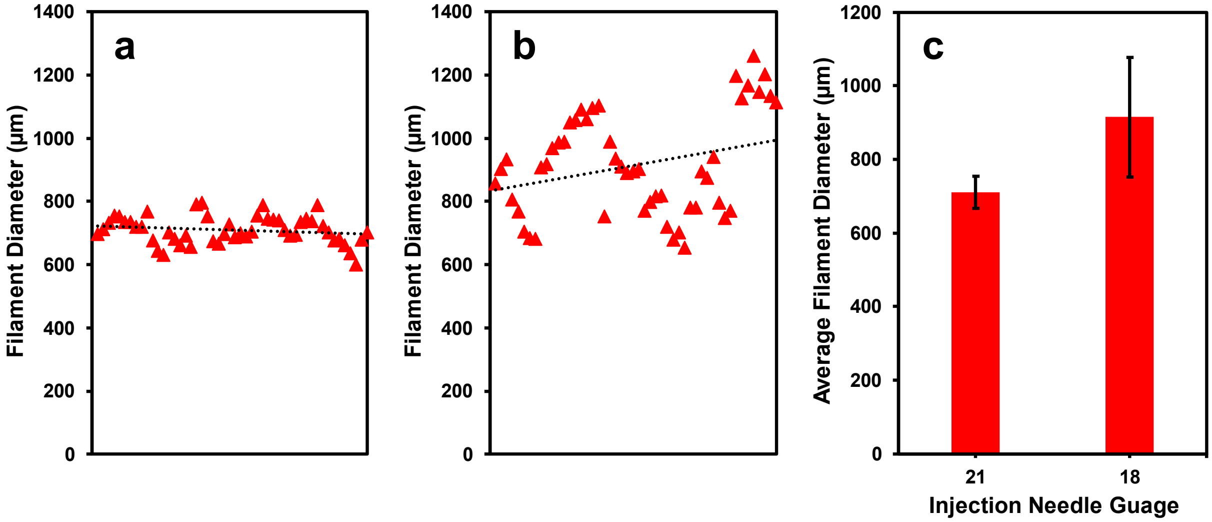

Figure S18: Filament diameter distribution of $Ti_3C_2T_x$/GO (20 wt% GO):GO Janus aerogels generated using needles with different gauge numbers of (a) 21 and (b) 18. (c) Average filament diameter of $Ti_3C_2T_x$/GO (20 wt% GO):GO Janus aerogels prepared by needles with different gauge numbers. All error bars represent the standard deviation.

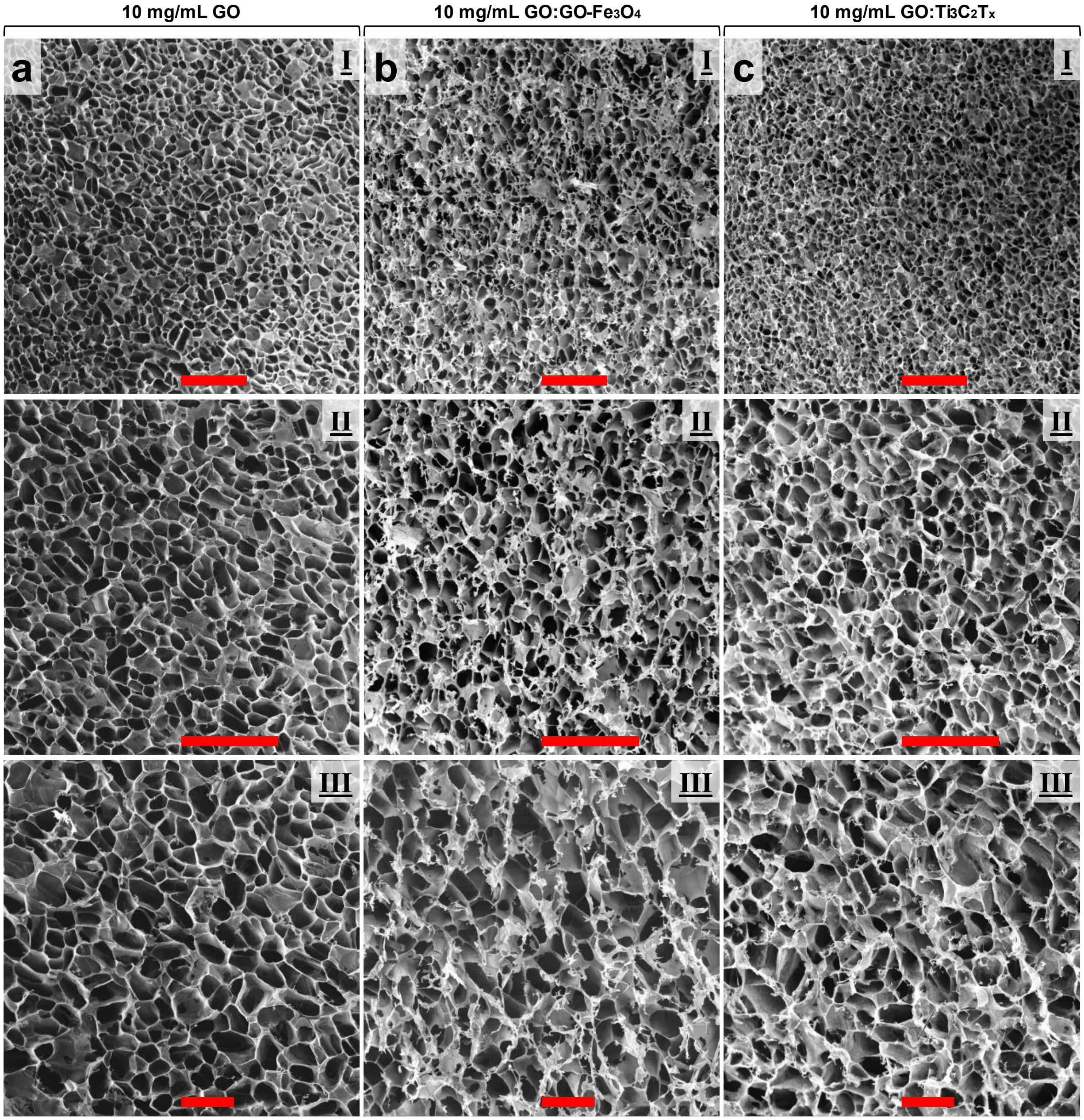

Figure S19: Micro-scale porosity of the hierarchical aerogels made of (a) 10 mg/ml GO, (b) 10 mg/ml mGO/GO (50 wt% GO), and (c)10 mg/ml $Ti_3C_2T_x$/GO (20 wt% GO) suspensions at (I-II) 500 µm, and (III) 200 µm scale bars.

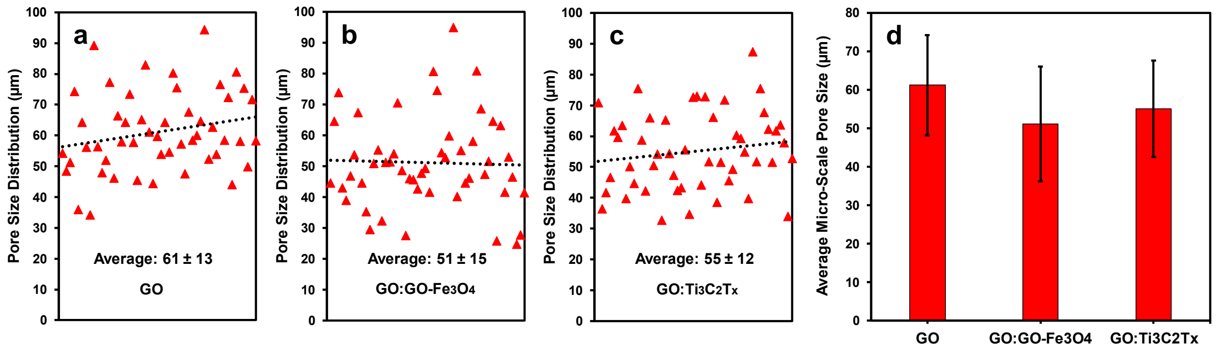

Figure S20: Micro-scale pore size distribution of aerogels made of (a) 10 mg/ml GO, (b) 10 mg/ml mGO/GO (50 wt% GO), and (c)10 mg/ml $Ti_3C_2T_x$/GO (20 wt% GO) suspensions. Average micro-scale porosities of generated aerogels out of 10 mg/ml GO, mGO/GO, and $Ti_3C_2T_x$/GO suspensions. All error bars represent the standard deviation.

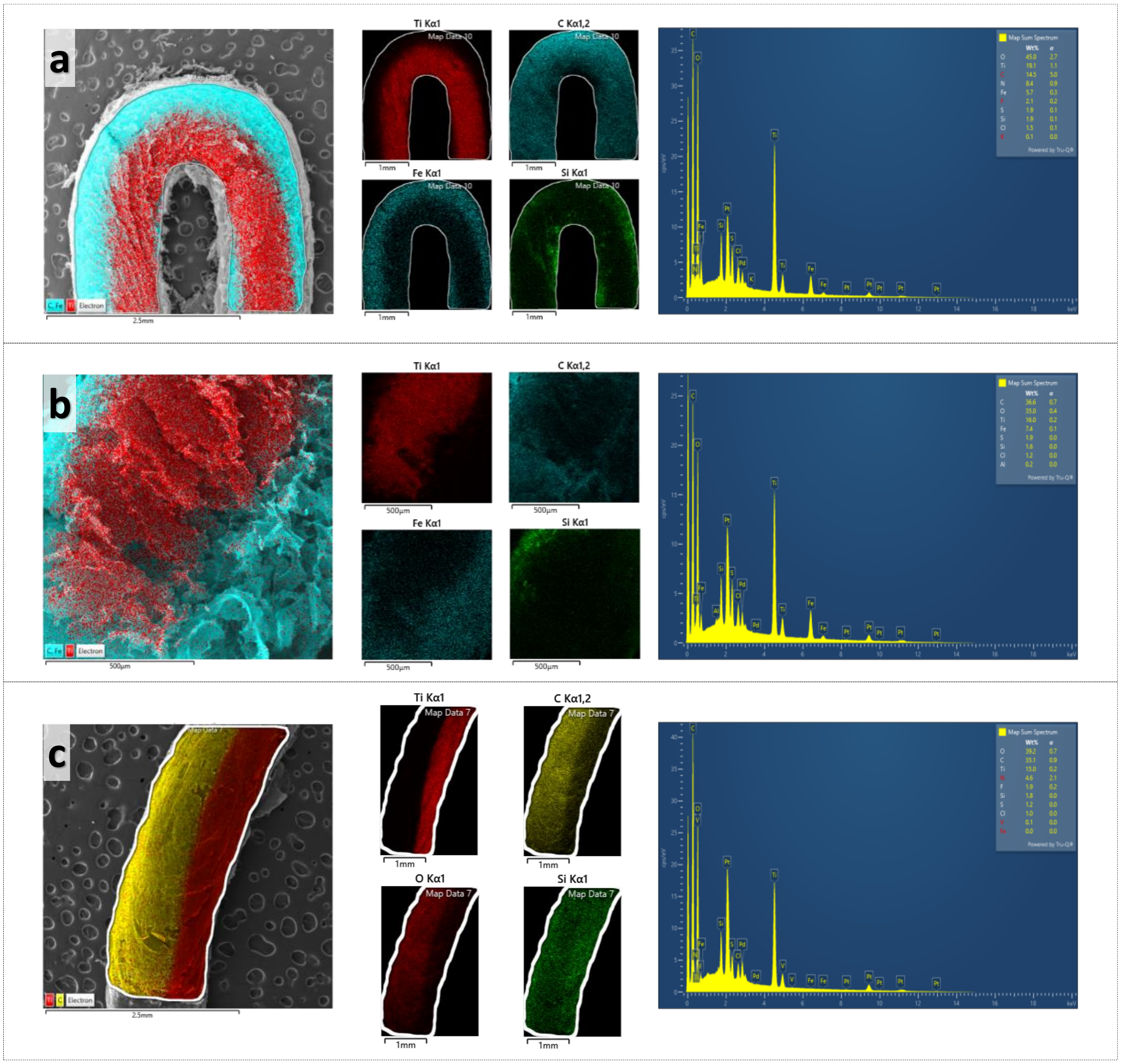

Figure S21: The EDX analysis of (a-b) $Ti_3C_2T_x$/GO:mGO/GO and (c) $Ti_3C_2T_x$/GO:GO Janus aerogels.

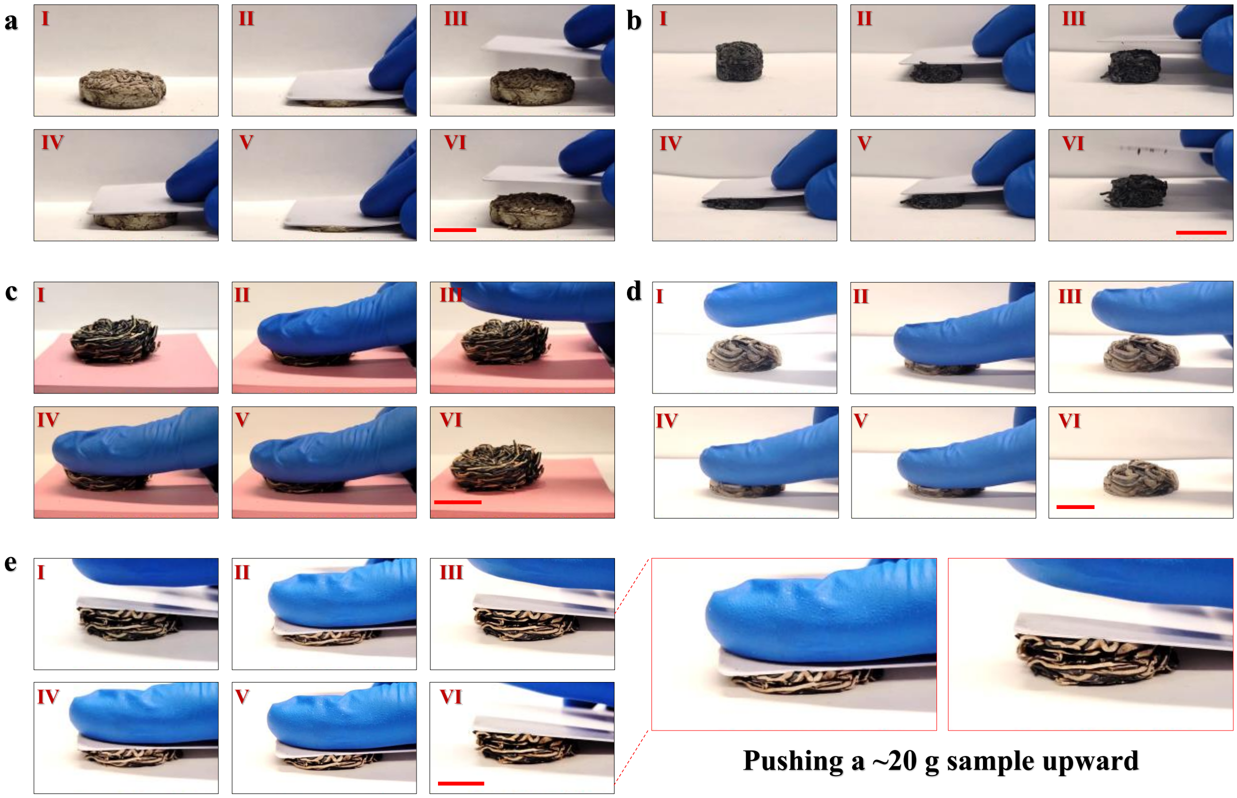

Figure S22: A comparison between the compressibility of various fabricated aerogels. (a) Worm-like aerogels of pure GO showcased elastic behavior, (b) while $Ti_3C_2T_x$/GO worm-like aerogels cannot elastically recover after compression. The Janus aerogels of (c, e) $Ti_3C_2T_x$/GO:mGO/GO and (d) $Ti_3C_2T_x$/GO:GO also showcased exceptional compressibility. (e) The Janus aerogel of $Ti_3C_2T_x$/GO:mGO/GO was capable of pushing a ~20 g sample upward after compression. All scale bars in (a-e) correspond to 2 cm.

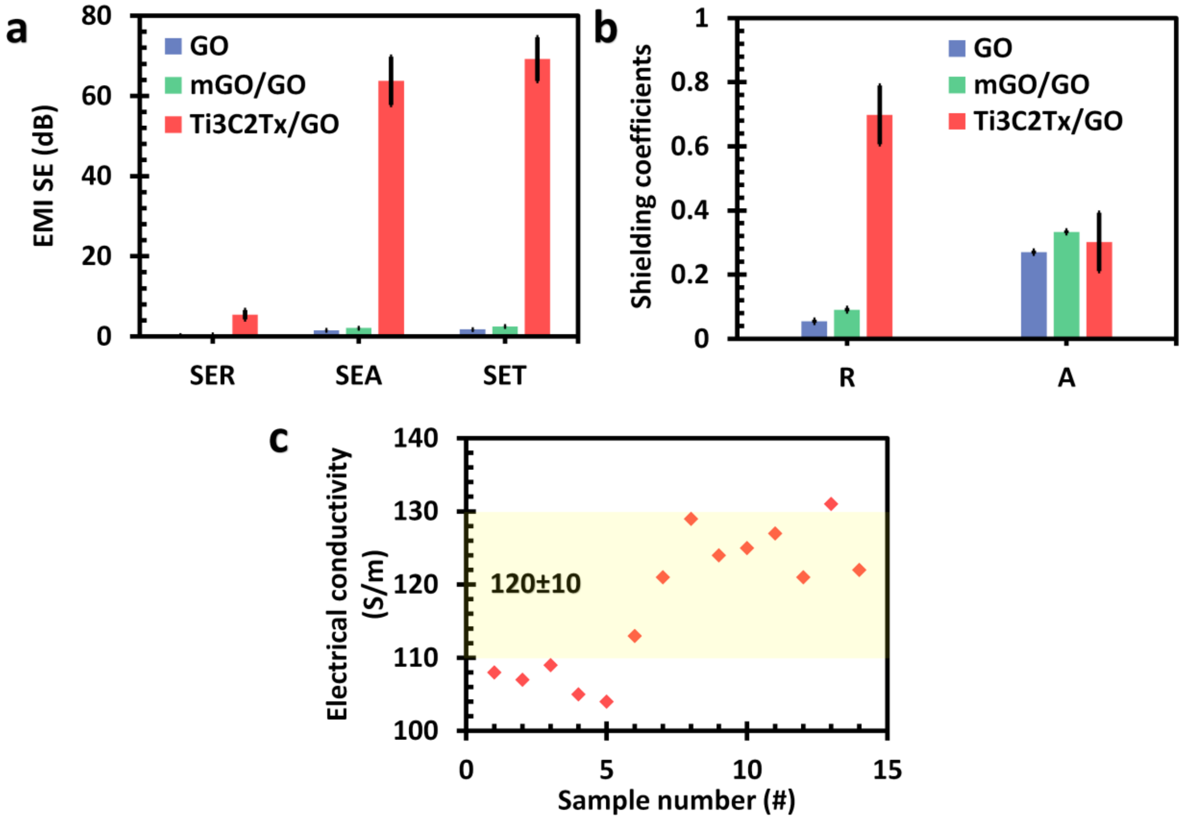

Figure S23: (a, b) EMI shielding characteristics of the worm-like structures composed of $Ti_3C_2T_x$/GO (20 wt% GO), mGO/GO (50 wt% GO), and pure GO. (c) Electrical conductivity of $Ti_3C_2T_x$/GO (20 wt% GO) worm-like aerogels. The thickness of these aerogels is 3mm. All the data pertaining to $Ti_3C_2T_x$/GO (20 wt% GO) are reproduced based on our previous open-access article released under the Creative Commons Attribution-NonCommercial License, which allows for the reproduction and reuse of the data upon proper citation and attribution.[40] All error bars represent the standard deviation.

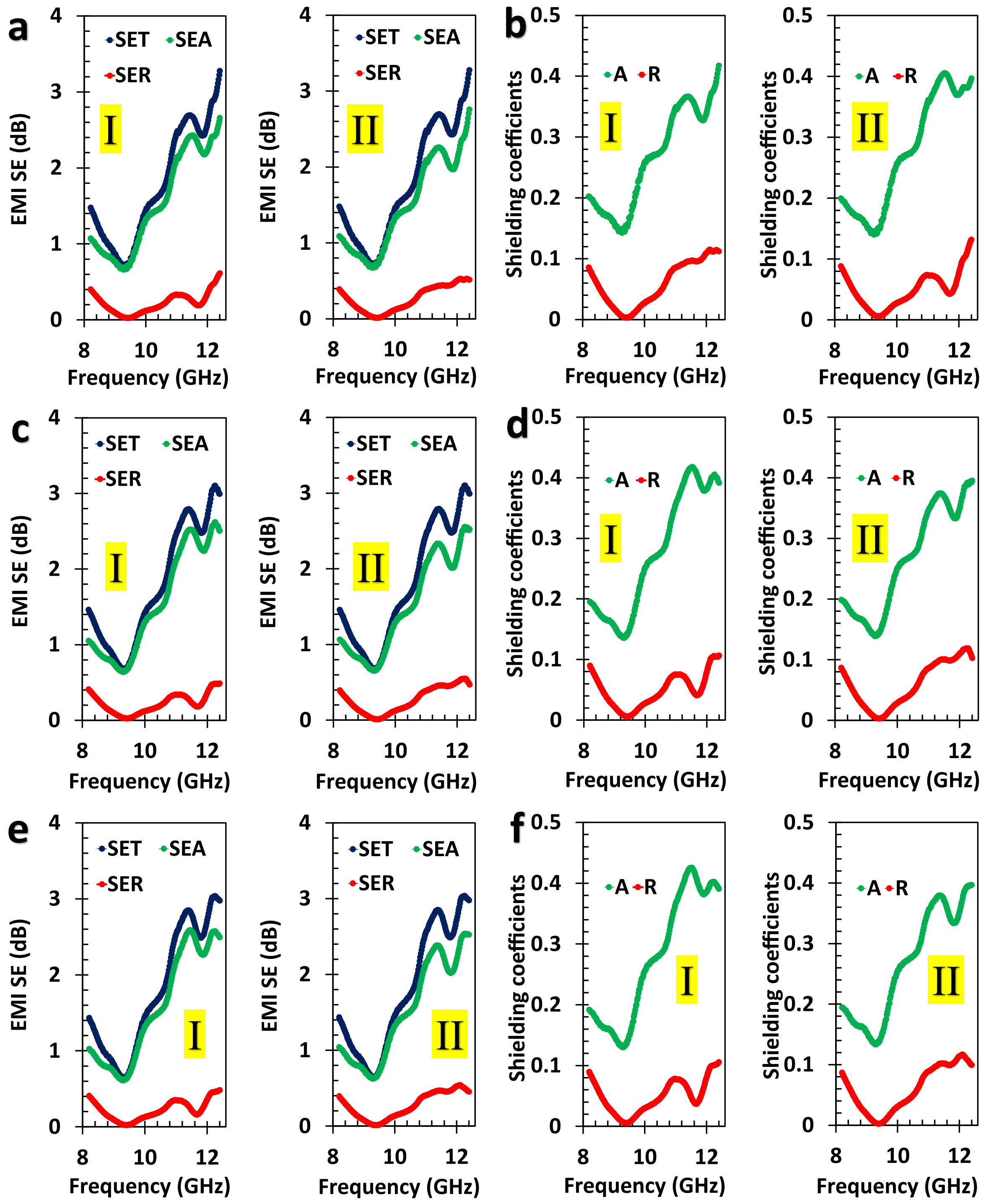

Figure S24: EMI shielding characteristics of pure GO aerogels, printed with a 200 μm nozzles. I and II refer to the data obtained from two different sides of the samples. The thickness of these samples was 3 mm. Three different samples were tested: (a, b) sample one, (c, d) sample two, and (e, f) sample three.

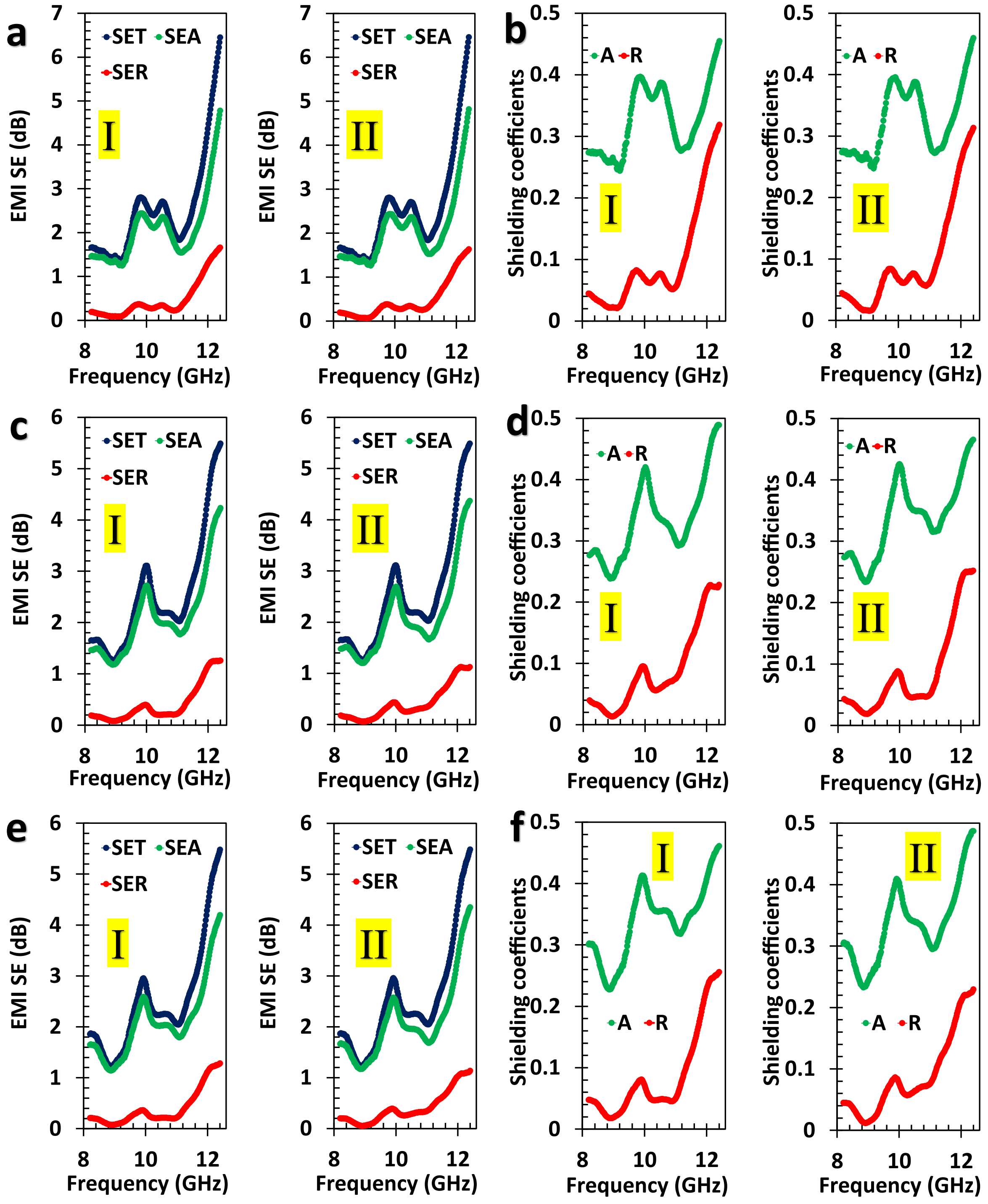

Figure S25: EMI shielding characteristics of mGO/GO (50 wt% GO) aerogels, printed with a 200 μm nozzles. I and II refer to the data obtained from two different sides of the samples. The thickness of these samples was 3 mm. Three different samples were tested: (a, b) sample one, (c, d) sample two, and (e, f) sample three.

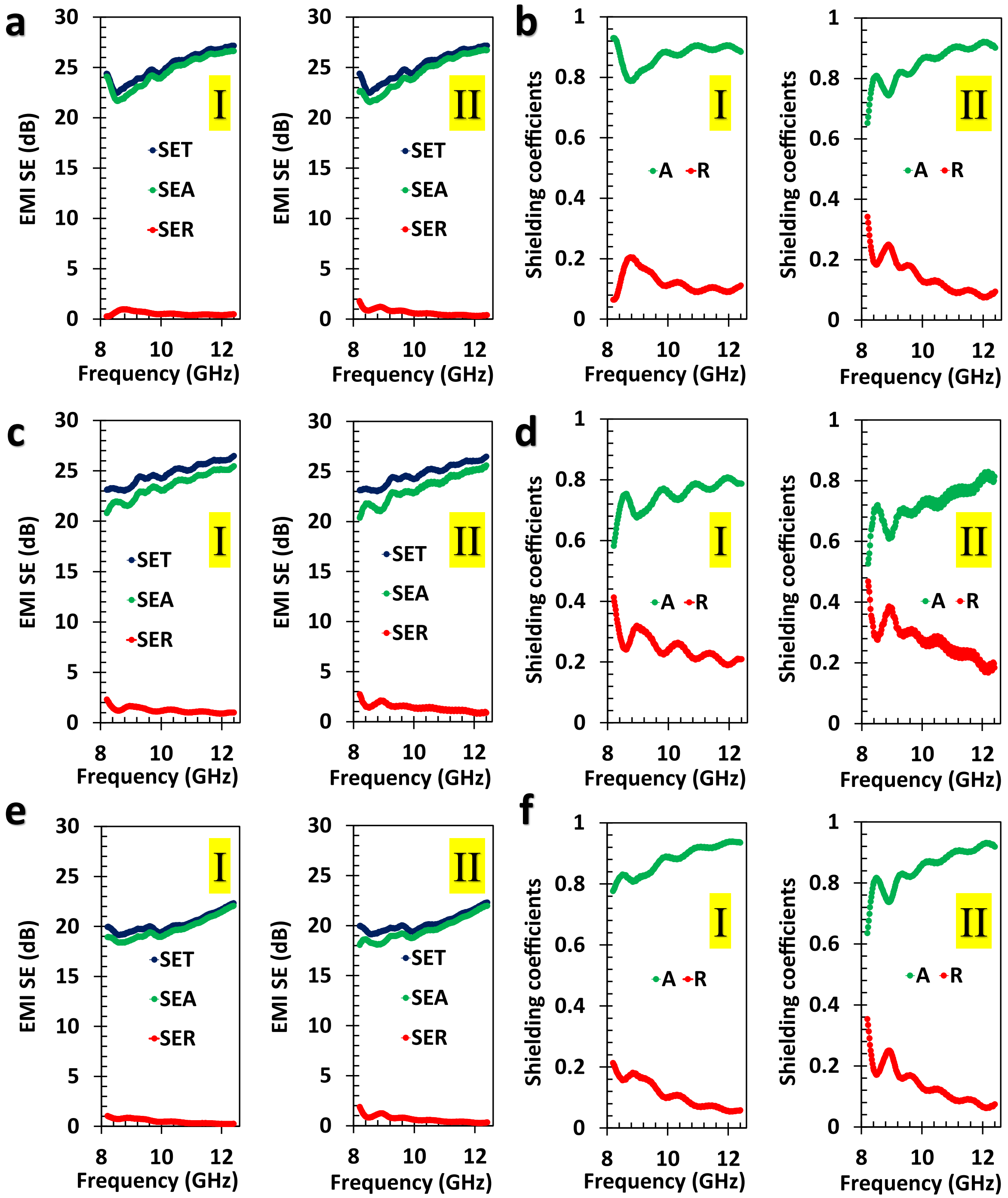

Figure S26: EMI shielding characteristics of $Ti_3C_2T_x$/GO (20 wt% GO) and mGO/GO (50 wt% GO) Janus aerogels, printed with two 200 µm nozzles. I and II refer to the data obtained from two different sides of the samples. The thickness of these samples was 5 mm. Three different samples were tested: (a, b) sample one, (c, d) sample two, and (e, f) sample three.

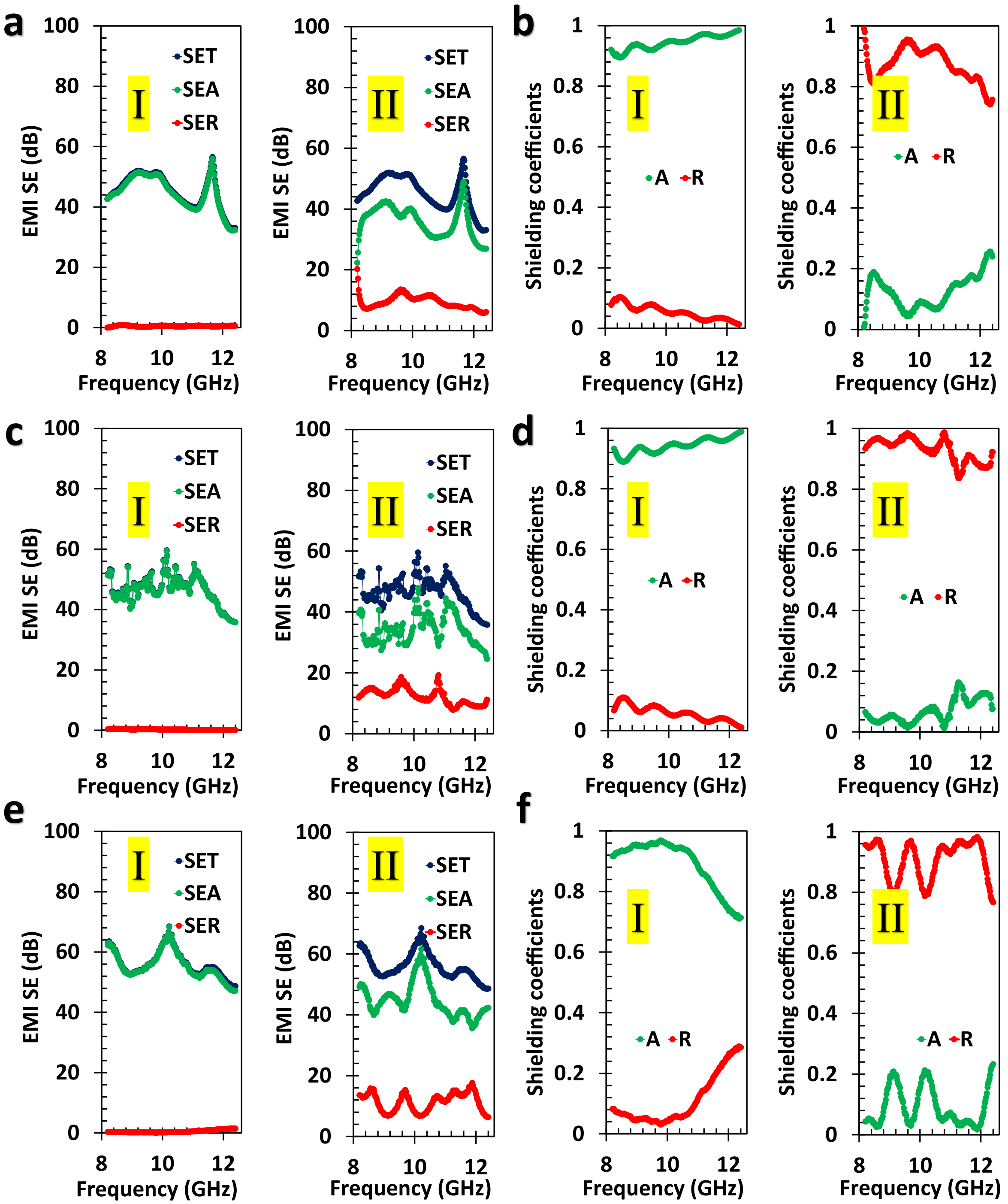

Figure S27: EMI shielding characteristics of copper coated magnetic/conductive Janus aerogels. This shield consists of a 3 mm Janus aerogel covered with 0.05 mm conductive copper tape from one side. The Janus aerogel of this shield is designed in a way that the magnetic parts' volume is larger than the conductive domain. I and II refer to the data obtained from two different sides of the samples: (I) Janus aerogel side and (II) copper side. Three different samples were tested: (a, b) sample one, (c, d) sample two, and (e, f) sample three.

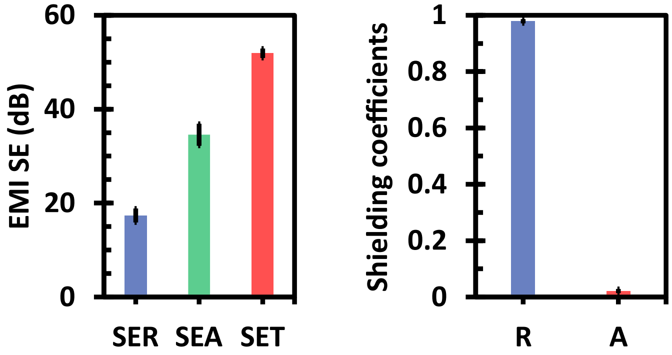

Figure S28: Average EMI shielding characteristics of 0.05 mm Cu tape. All error bars represent the standard deviation.

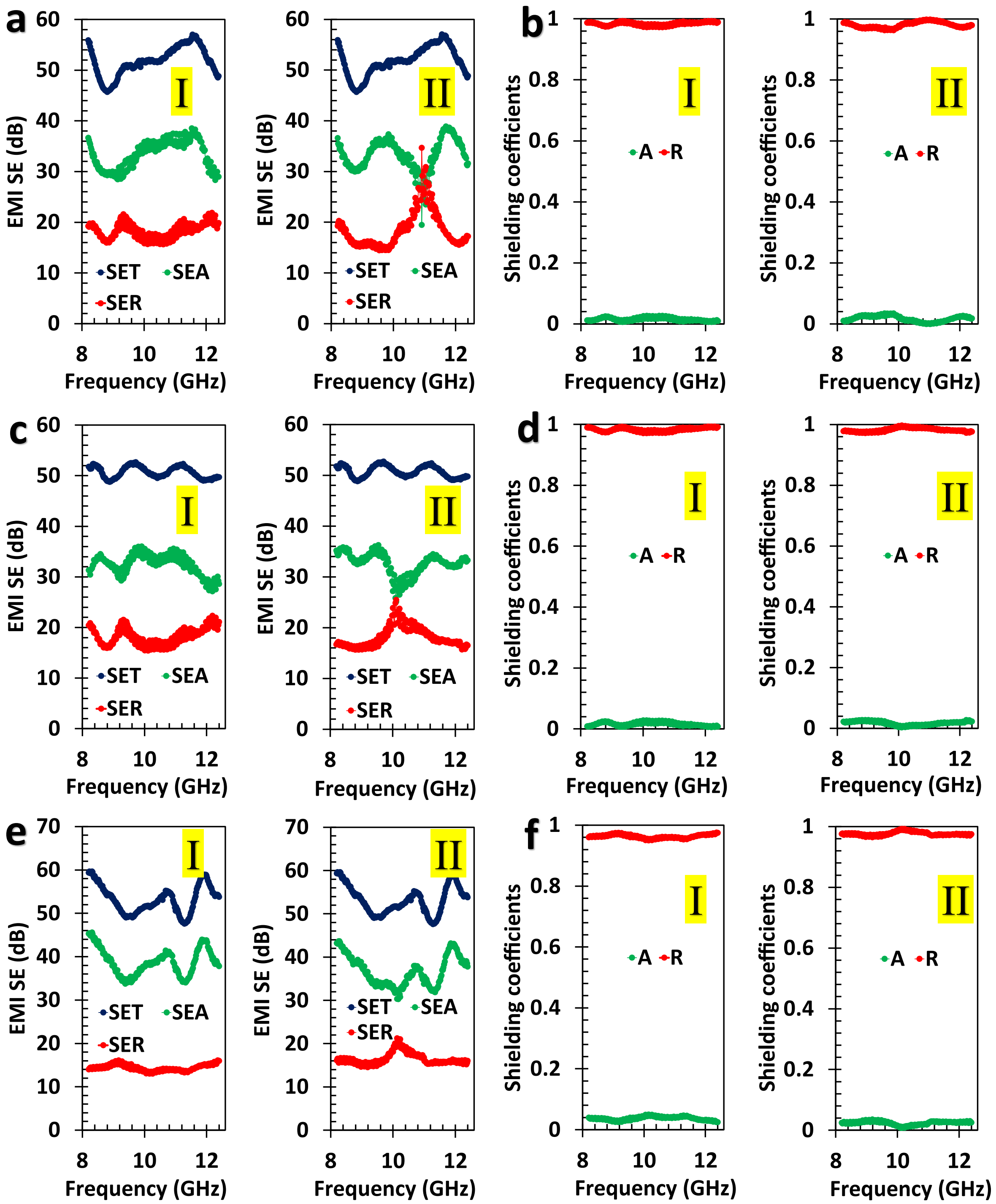

Figure S29: EMI shielding characteristics of 0.05 mm Cu tape. Three different sections of the tape were tested: (a, b) sample one, (c, d) sample two, and (e, f) sample three.

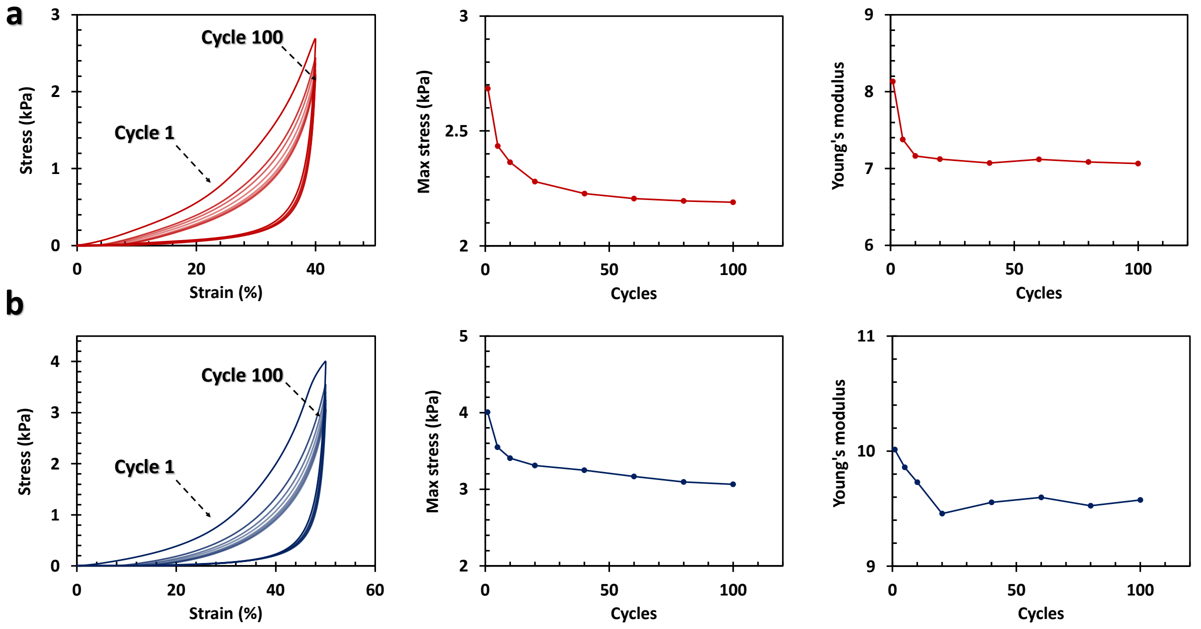

Figure S30: Fatigue hysteresis tests of $Ti_3C_2T_x$/GO (20 wt% GO) and GO Janus aerogels. The samples were compressed up to (a) 40% and (b) 50% strain for 100 cycles. The results showed minor plastic deformation at those strain levels, where the aerogels maintained ~80% of their initial Young's moduli and maximum stresses.

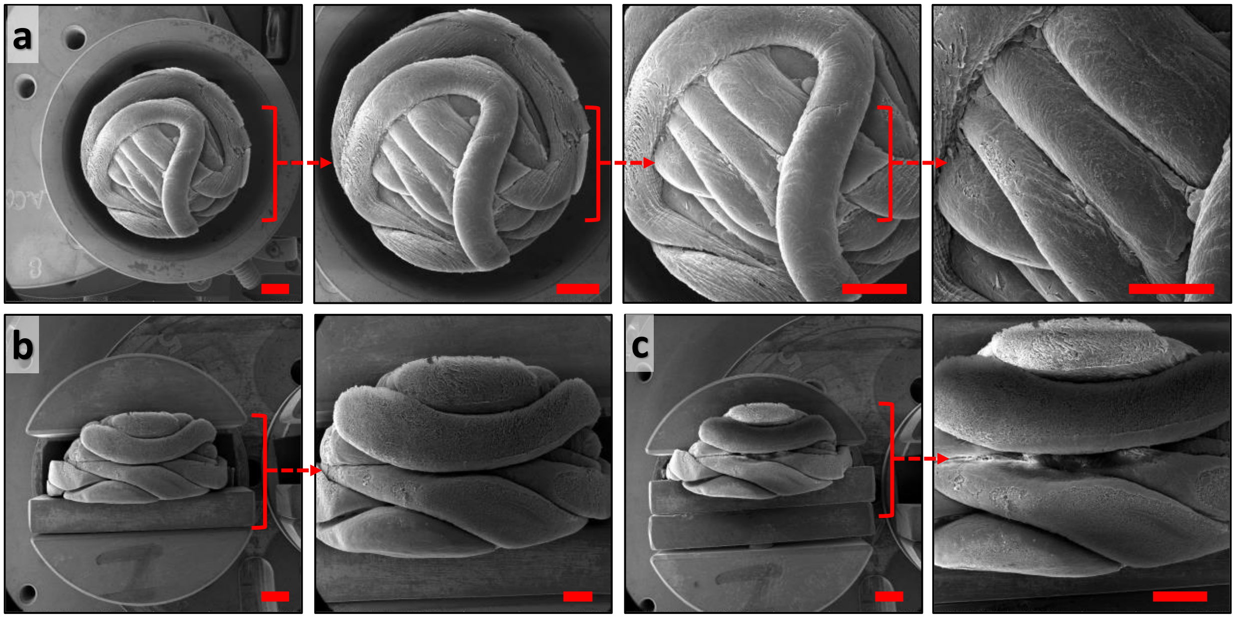

Figure S31: SEM images of Janus aerogels of $Ti_3C_2T_x$/GO (20 wt% GO) and GO after 100 compression cycles. The sample shown in (c) was compressed during SEM imaging. All scale bars in (a-c) correspond to 1 mm.

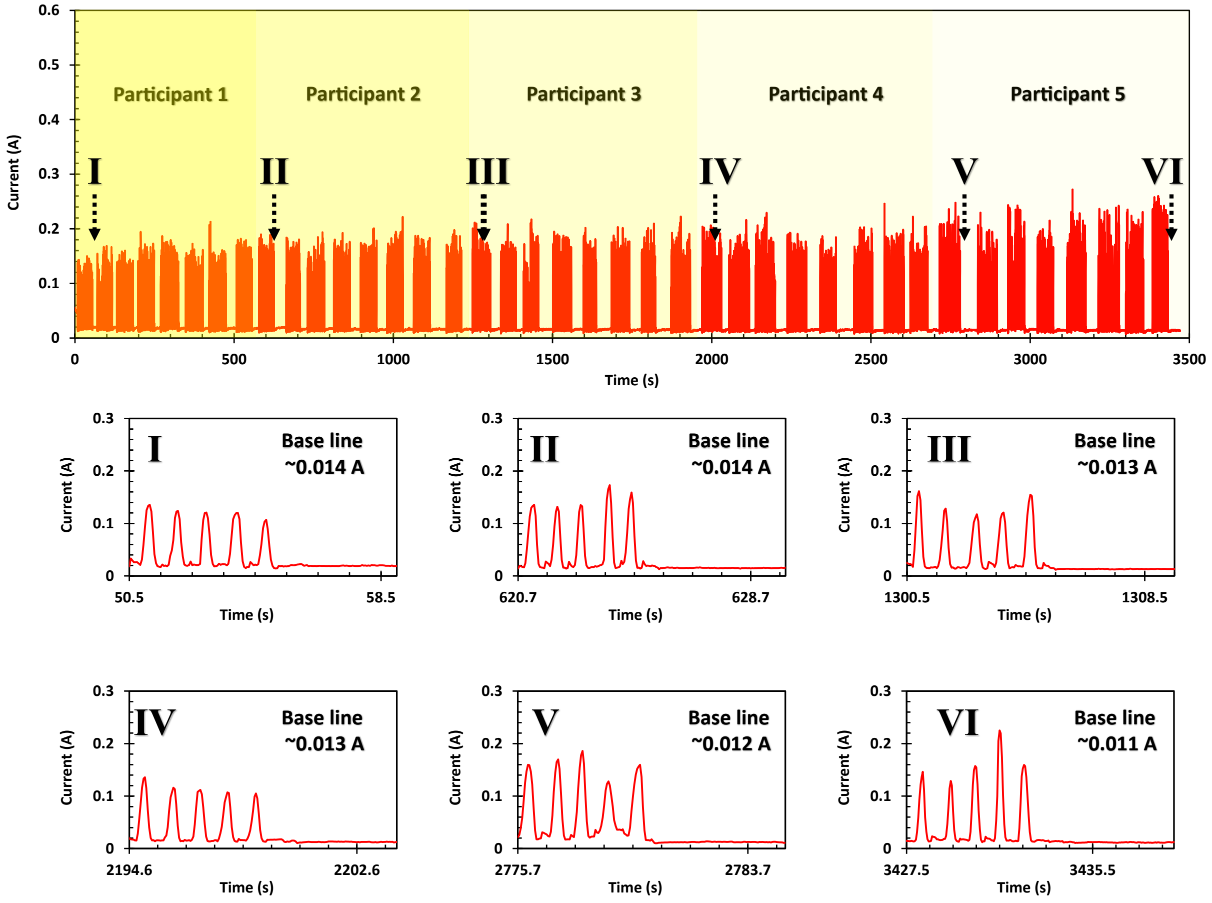

Figure S32: The durability of the sensor after 2,000 compression cycles at real conditions. To assess the durability of these sensors, five different participants were asked to touch the sensor, and its response was recorded over the course of the experiment.

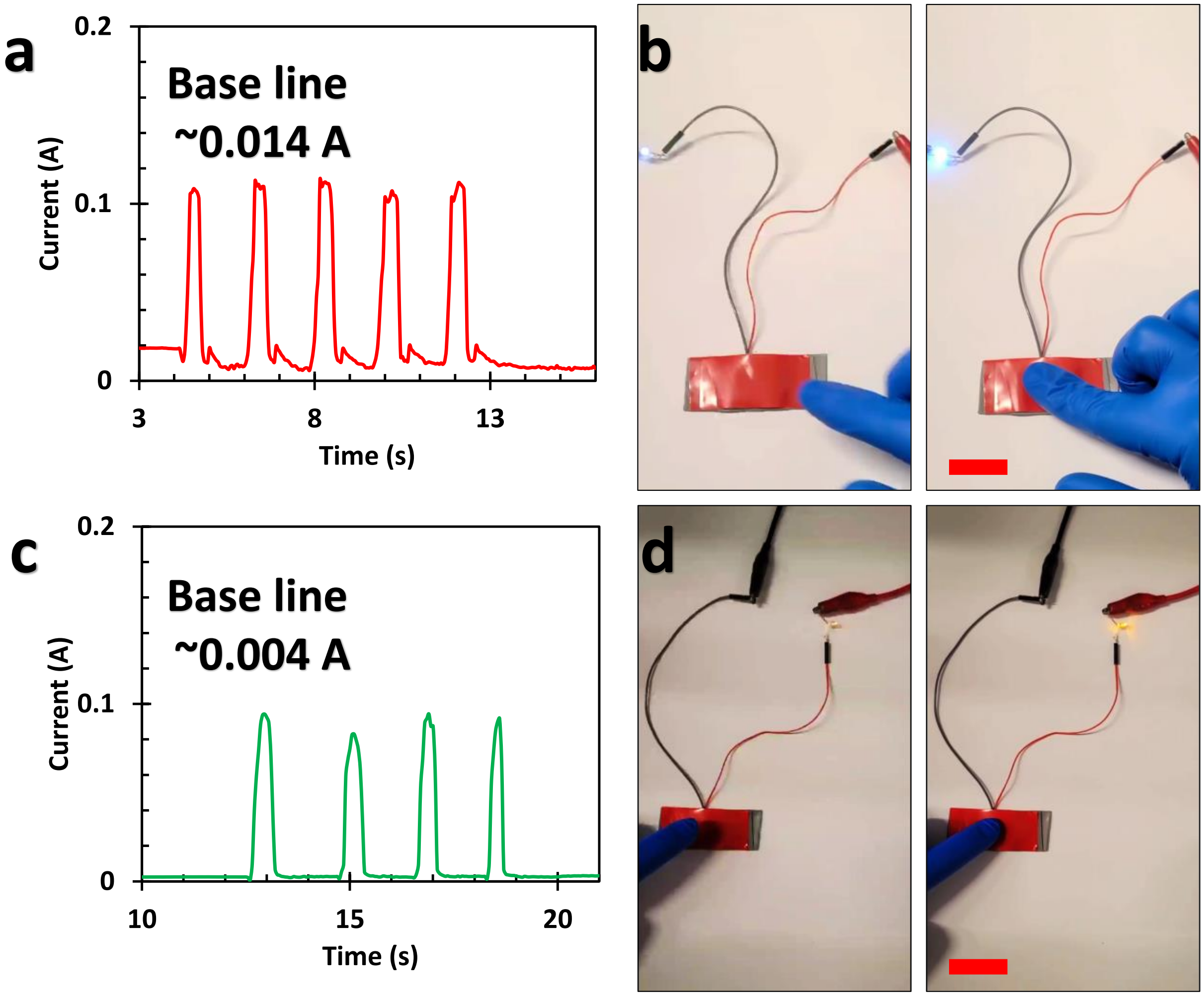

Figure S33: The recorded response and digital images of the sensing performance of the fabricated structures (a-b) before and (c-d) after three months. All scale bars in (b, d) correspond to 2 cm.

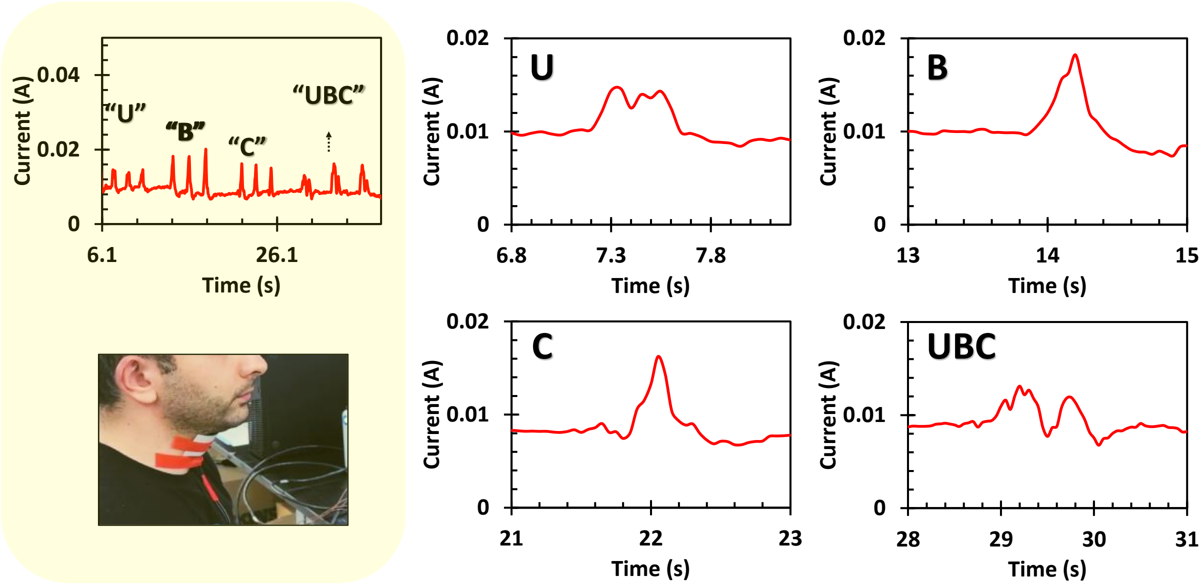

Figure S34: The capability of the fabricated device to distinguish different alphabets and even polysyllabic words when attached to the skin outside of the larynx of a human subject.

5. Tables

Table S1: A comparison of the SE_T vs. A of the magnetic/conductive Janus structures with the previous best practices in the literature. In the case that R and A coefficients were not directly reported in the papers, these coefficients are calculated based on the formulations explained in section three of this file.

Sample	Thickness (mm)	EMI SE (dB)	SE_R (dB)	R	A	Ref.
CNTs–multilayered graphene edge plane core–shell hybrid foams	1.6	38.4	3.9	0.59	0.41	[41]
$Ti_3C_2T_x$	0.001	46.1	13.5	~0.96	~0.04	[42]
3D graphene fibers	0.0263	56	~9	~0.89	0.11	[43]
rGO film	0.0084	20	~8	~0.84	~0.16	[44]
Glucose-derived anisotropically oriented carbon films (GCFs)	0.00048	21.72	7.96	0.84	0.16	[45]
$Ti_3C_2T_x$/PS[a]	2	61.2	6.5	~0.78	~0.22	[46]
MWCNTs/WPU[b] foam	2.3	50.5	~9	~0.89	~0.11	[47]
PCC[c]/MXene/PVA[d] phase change composite film	0.048	43.1	13.17	~0.96	~0.04	[48]
CNTs sponge/epoxy	2	44	5	~0.68	~0.32	[49]
SWCNTs/ $Ti_3C_2T_x$ /latex	1.1	52	~19	~0.98	~0.02	[50]
ANFs[e] reinforced PVA hydrogels and a layer of AgNWs[f]/PVA	~0.3	52	16	~0.97	~0.03	[51]
Pristine $Ti_3C_2T_x$	0.014	82	~20	~0.99	~0.01	[52]
Wrinkled $Ti_3C_2T_x$	0.02	68	27	0.99>	<0.01	[53]
AgNWs decorated leather	0.5	55	~8	~0.84	~0.16	[54]
$Ti_3C_2T_x$ aerogel/epoxy	2	34.5	~6.5	~0.78	~0.22	[55]
Fe_3O_4/PVA composite electrospun nanofibers in the top and bottom layers and $Ti_3C_2T_x$ /PVA composite electrospun nanofibers in the middle layer	0.075	40	~13	~0.96	0.04	[56]
AgNWs wrapped carbon hybrid sponge	3	70.1	~11	~0.91	~0.09	[57]
MWCNTs/Fe_3O_4@Ag/ Epoxy	2	35	4.5	0.65	0.35	[58]
Graphene /Fe_3O_4 papers	0.25	24	8	0.84	0.16	[59]
MWCNTs/PLLA[g]	1.5	30	4	0.6	0.4	[60]
CNTs/GTR[h]	1	30	6	0.75	0.25	[61]
Fe_3O_4@rGO/PMMA[i]	2.9	29.3	4	0.6	0.4	[62]
rGO foam	0.9	42.3	10	0.9	0.1	[63]
MWCNTs/PVDF	1	22.5	6.3	0.77	0.23	[64]
Graphene /MWCNTs/PMMA	2	36	6.67	0.78	0.22	[65]
CNTs/rGO foam	2	31.2	5.9	0.74	0.26	[66]
Multilayer Fe_3O_4@rGO/MWCNTs	0.8	36	1.46	0.27	0.73	[67]

/WPU						
Multilayer $Ti_3C_2T_x$/CNF[j]	0.035	39.6	17.7	0.98	0.02	68
rGO/PDMS	5	26	2.99	0.49	0.51	7
Ti_2CT_x/PVA	0.3	21	11	0.91	0.09	69
Ti_2CT_x/PVA foam	5	28	2	0.37	0.63	69
$Ti_3C_2T_x$/PEDOT:PSS[k] aerogels	5	28	3	0.5	0.5	70
$Ti_3C_2T_x$/ polyaniline/ carbon fiber	0.55	26.5	5.7	0.74	0.26	71
Multilayer $Ti_3C_2T_x$/PVA	0.027	44.4	8.3	0.85	0.15	72
$Ti_3C_2T_x$/ANF	0.018	55.9	10.3	0.9	0.1	73
Polyaniline-Grafted $Ti_3C_2T_x$/PVDF	1.7	33.3	5.2	0.7	0.3	74
Silver/electrospun nylon nanofiber	0.005	77.6	13	~0.95	~0.5	75
FGQF[l]	0.2	~48	~9.6	~0.89	~0.11	76
FGQF	1.12	106	~10	~0.9	~0.1	76
Nacre-like graphene/PI[m]	0.01	43.8	~13.3	~0.95	~0.05	77
MXene-functionalized PEDOT:PSS[m] hydrogel	0.295	51.7	3.7	0.58	0.42	78
$Ti_3C_2T_x$/MWCNTs/$SrFe_{12}O_{19}$	0.04	62.9	~12	~0.94	~0.06	79
Ionic liquid doped PEDOT:PSS hydrogel	0.046	54	~18.4	~0.98	<0.02	80
Screen-printed $Ti_3C_2T_x$/Xanthan Gum hybrids	0.012	40.1	~15	~0.97	~0.03	81
Cu NWs/graphene core–shell aerogels	5	43.7	2.86	~0.48	~0.52	82
Compressed Cu NWs /graphene core–shell aerogels	5	31.28	4.11	~0.61	~0.39	82
MWCNTs/WPU	2.3	46.7	~7.2	~0.8	~0.2	83
$Ti_3C_2T_x$/gelation	0.004	53.6	~19.2	~0.98	<0.02	84
$Ti_3C_2T_x$	0.005	57.4	17.5	~0.98	~0.02	85
Ti_3CNT_x	0.005	49.7	17	~0.98	~0.02	85
Honeycomb porous graphene/AgNWs	0.0483	61.8	~14.5	~0.96	~0.04	86
2D Cu nanosheets	0.063	105	~14	~0.96	~0.04	87
AgNWs/ Nanocellulose aerogels	2	70.5	~17	~0.98	~0.02	88
CNTs/ANFs hybrid aerogel	0.568	54.4	~8	~0.84	~0.16	89
Polyolefin Composites with a $Ti_3C_2T_x$/graphene framework	1.85	61	7.7	0.83	0.17	90
$Ti_3C_2T_x$/CNF	0.074	25.8	5.3	~0.71	~0.29	91
$Ti_3C_2T_x$/polydopamine hybrids	0.007	58.4	~16	~0.97	~0.03	92
Graphite film	~0.0004	27.8	~5	~0.69	~0.31	93
SrGO/$(C\text{-}SiC)_{12}$	4	70.2	~12	~0.94	~0.06	94
rGO wrapped AgNWs networks	~0.0001	35	8.1	~0.84	~0.16	95
$Ti_3C_2T_x$/rGO aerogel	2	56.4	5.7	0.73	0.27	96
Laminated ANF/CNTs	0.024	36	~14	~0.96	~0.04	97
$Ti_3C_2T_x$ /ANF	0.037	48	~18	~0.98	~0.02	98
Densified CNTs film	~0.015	101	~20	~0.99	~0.01	99
CF[n]-$NiCo_2O_4$–4F	0.034	53	~8.2	0.85	0.15	100
CF-NiCo	0.034	45	~10	0.90	0.1	100
CF	0.032	45	~16	0.97	0.03	100

Polydopamine-modified expanded graphite/PI	0.5	101	~19	~0.98	~0.02	101
Recycled carbon fibers/ $Ti_3C_2T_x$	0.343	45.7	~15	~0.97	~0.03	102
Carbonized wood/Ni/CNTs	-	48.2	2.8	~0.47	~0.53	103
Ti3C2Tx/ magnetic GO Janus aerogel	5	23.4	0.83	0.17	0.83	This study
Ti3C2Tx/ magnetic GO Janus aerogel coated with cu tape	3	51	0.4	0.089	0.91	This study

[a]polystyrene, [b]water-borne polyurethane, [c]phase change capsules, [d]polyvinyl alcohol, [e]Aramid nanofibers, [f]silver nanowires, [g]poly(L-lactide), [h]ground tire rubber, [i]poly (methyl methacrylate), [j]cellulose nanofiber, [k]poly(3,4-ethylenedioxythiophene) polystyrene sulfonate, [l]ferromagnetic graphene quartz fiber, m polyimide, [n]Carbon fabric

Table S2: A comparison between the main characteristics of our sensor and those structures reported in the previous best practices in the literature.

Material	Sensitivity/GF	Detection range	Stability	Preparation method	Ref
rGO aerogel	0.46 kPa^{-1}	0.5–8 kPa	4200	Solvothermal (Chemical reduction) and thermal annealing	104
N-doped rGO sponge	1.33 kPa^{-1}	30%-40% compression	3000	Hydrothermal (Chemical reduction and doping) and thermal annealing	105
rGO sponge	0.79–1.46	50% compression	500	Emulsion assembly alongside chemical reduction	106
rGO aerogel	-	up to 90% compression	-	3D printing and thermal annealing	107
Biomimetic rGO aerogel	-	0–50%	6000	Bidirectional freezing technique and thermal annealing	108
MoS_2/graphene/Ecoflex	6.06 kPa^{-1}	-	4000	Growing graphene on Ni foam, immersing in Ecoflex, and etching the Ni template	109
RGO-PU[a]	0.26 kPa^{-1}	0–10 kPa	10,000	Dip coating PU foam in GO suspension and hydrothermal reduction	110
3D graphene–PDMS[b] hollow structure	15.9 kPa^{-1}	-	-	Growing graphene on Ni foam, immersing in PDMS, and etching the Ni template	111
rGO paper	17.2 kPa^{-1}	0–20 kPa	300	Immersing a paper in GO suspension and thermal reduction	112
Wood-derived carbon aerogel	5.16 kPa^{-1}	0–16.89 kPa	1000	Carbonization of alkali lignin/CNF[c] aerogel	113
Hollow $Ti_3C_2T_x$ spheres/rGO aerogel	609 kPa^{-1}	0-10 kPa	6000	Fabrication of PS[d] microspheres, electrostatic self-assembly of $Ti_3C_2T_x$ on PS, mixing with go suspension, and thermal reduction and removing the PS spheres via annealing	114

$Ti_3C_2T_x$/CNC[e] aerogel	114.6 kPa^{-1}	50 Pa–10 kPa	10000	Freeze casting, annealing, and carbonization	115
$Ti_3C_2T_x$/ carboxyated carbon nanotubes/ carboymethyl chitosan	0.18-3.84 kPa^{-1}	0–12.4 kPa-32.8–80.9 kPa	1000	Freeze casting and dipping in $Ti_3C_2T_x$	116
$Ti_3C_2T_x$/carbon nanofiber	65 kPa^{-1}	up to 95% compression	5000	Freeze casting	117
$Ti_3C_2T_x$/rGO/PI[f] aerogel	0.08 kPa^{-1}	0-180 kPa	10000	Freeze-drying and thermal reduction	118
PI nanofiber / $Ti_3C_2T_x$ composite aerogel	0.002-0.014 kPa^{-1}	up to 90% compression	1000	PI nanofiber was first treated with O_2 plasma, mixed with $Ti_3C_2T_x$, and then freeze dried to form aerogel. The prepared aerogel was then annealed at high temperature.	119
$Ti_3C_2T_x$/rGO	4.05-22.56 kPa^{-1}	1 - 3.5 kPa	10000	Freeze-drying and thermal reduction	120
RGo/PVA[g]	1.32-7.48 kPa^{-1}	0-3 kPa	1000	Bidirectional freezing and thermal reduction.	121
Wave-layered carbon aerogels	10.08 kPa^{-1}	0-10 kPa	3000	A mixture of glucose and dicyandiamide was first carbonized into multi-layered nanosheets and then dispersed in CNF suspension. A multi-step processing, including directional freeze-casting, freeze-drying, and carbonization was used to prepare the carbon aerogels.	122
$Ti_3C_2T_x$/GO Janus aerogel	1-8.18 kPa^{-1}	0~11 kPa	2000 at real conditions	Liquid-liquid sculpting without any freeze casting or thermal/chemical reduction	This study

Data declaration and availability

All data generated in this study have been securely deposited on Figshare and are openly accessible at the following DOI: https://doi.org/10.6084/m9.figshare.24455059. All SEM images, including the original scale bar and information, can be found in raw data.

References

1. Marcano DC, Kosynkin DV, Berlin JM, Sinitskii A, Sun Z, Slesarev A*, et al.* Improved synthesis of graphene oxide. *ACS nano* 2010, **4**(8)**:** 4806-4814.

2. Hashemi SA, Mousavi SM, Faghihi R, Arjmand M, Rahsepar M, Bahrani S*, et al.* Superior X-ray radiation shielding effectiveness of biocompatible polyaniline reinforced with hybrid graphene oxide-iron tungsten nitride flakes. *Polymers* 2020, **12**(6)**:** 1407.

3. Hashemi SA, Mousavi SM, Naderi HR, Bahrani S, Arjmand M, Hagfeldt A*, et al.* Reinforced polypyrrole with 2D graphene flakes decorated with interconnected nickel-tungsten metal oxide complex toward superiorly stable supercapacitor. *Chemical Engineering Journal* 2021, **418:** 129396.

4. Hashemi SA, Naderi HR, Mousavi SM, Bahrani S, Arjmand M, Dimiev AM*, et al.* Synergic effect of laser-assisted graphene with silver nanowire reinforced polyindole/polypyrrole toward superior energy density. *Carbon* 2022, **188:** 276-288.

5. Kamkar M, Erfanian E, Bazazi P, Ghaffarkhah A, Sharif F, Xie G*, et al.* Interfacial Assembly of Graphene Oxide: From Super Elastic Interfaces to Liquid-in-Liquid Printing. *Adv Mater Interfaces* 2022, **9**(6)**:** 2101659.

6. Amini M, Kamkar M, Rahmani F, Ghaffarkhah A, Ahmadijokani F, Arjmand M. Multilayer Structures of a Zn0. 5Ni0. 5Fe2O4-Reduced Graphene Oxide/PVDF Nanocomposite for Tunable and Highly Efficient Microwave Absorbers. *ACS Applied Electronic Materials* 2021.

7. Kamkar M, Ghaffarkhah A, Ajdary R, Lu Y, Ahmadijokani F, Mhatre SE*, et al.* Structured Ultra-Flyweight Aerogels by Interfacial Complexation: Self-Assembly Enabling Multiscale Designs. *Small* 2022**:** 2200220.

8. Ghaffarkhah A, Kamkar M, Dijvejin ZA, Riazi H, Ghaderi S, Golovin K*, et al.* High-resolution extrusion printing of Ti3C2-based inks for wearable human motion monitoring and electromagnetic interference shielding. *Carbon* 2022, **191:** 277-289.

9. Shuck CE, Sarycheva A, Anayee M, Levitt A, Zhu Y, Uzun S*, et al.* Scalable Synthesis of Ti3C2Tx MXene. *Advanced Engineering Materials* 2020, **22**(3)**:** 1901241.

10. Sarycheva A, Gogotsi Y. Raman spectroscopy analysis of the structure and surface chemistry of Ti3C2T x MXene. *Chemistry of Materials* 2020, **32**(8)**:** 3480-3488.

11. Hu T, Hu M, Gao B, Li W, Wang X. Screening surface structure of MXenes by high-throughput computation and vibrational spectroscopic confirmation. *The Journal of Physical Chemistry C* 2018, **122**(32)**:** 18501-18509.

12. Lioi DB, Neher G, Heckler JE, Back T, Mehmood F, Nepal D*, et al.* Electron-Withdrawing Effect of Native Terminal Groups on the Lattice Structure of Ti3C2T x MXenes Studied by Resonance Raman Scattering: Implications for Embedding MXenes in Electronic Composites. *ACS Applied Nano Materials* 2019, **2**(10)**:** 6087-6091.

13. Zhang T, Zhang W, Xi H, Li Q, Shen M, Ying G*, et al.* Polydopamine functionalized cellulose-MXene composite aerogel with superior adsorption of methylene blue. *Cellulose* 2021, **28**(7)**:** 4281-4293.

14. Li Y, Zhou X, Wang J, Deng Q, Li M, Du S*, et al.* Facile preparation of in situ coated Ti 3 C 2 T x/Ni 0.5 Zn 0.5 Fe 2 O 4 composites and their electromagnetic performance. *Rsc Advances* 2017, **7**(40)**:** 24698-24708.

15. Maleski K, Ren CE, Zhao M-Q, Anasori B, Gogotsi Y. Size-dependent physical and electrochemical properties of two-dimensional MXene flakes. *ACS applied materials & interfaces* 2018, **10**(29)**:** 24491-24498.

16. Hashemi SA, Bahrani S, Mousavi SM, Omidifar N, Behbahan NGG, Arjmand M*, et al.* Antibody mounting capability of 1D/2D carbonaceous nanomaterials toward rapid-specific detection of SARS-CoV-2. *Talanta* 2022, **239:** 123113.

17. Ossonon BD, Bélanger D. Synthesis and characterization of sulfophenyl-functionalized reduced graphene oxide sheets. *RSC advances* 2017, **7**(44)**:** 27224-27234.

18. Hashemi SA, Bahrani S, Mousavi SM, Omidifar N, Arjmand M, Lankarani KB*, et al.* Differentiable detection of ethanol/methanol in biological fluids using prompt graphene-based electrochemical nanosensor coupled with catalytic complex of nickel oxide/8-hydroxyquinoline. *Analytica Chimica Acta* 2022, **1194:** 339407.

19. Covarrubias-García I, Quijano G, Aizpuru A, Sánchez-García JL, Rodríguez-López JL, Arriaga S. Reduced graphene oxide decorated with magnetite nanoparticles enhance biomethane enrichment. *Journal of hazardous materials* 2020, **397:** 122760.

20. Goodarzi M, Pircheraghi G, Khonakdar HA. Tailoring the graphene polarity through the facile and one-step electrochemical exfoliation in low concentration of exfoliation agents. *FlatChem* 2020, **22:** 100181.

21. Goodarzi M, Pircheraghi G, Khonakdar HA, Altstadt V. Flexible high dielectric polystyrene/ethylene-α-octene copolymer/graphene nanocomposites: Tuning the morphology

and dielectric properties by graphene's surface polarity. *Polymers for Advanced Technologies* 2022, **33**(3)**:** 937-951.

22. Ghaffarkhah A, Kamkar M, Riazi H, Hosseini E, Dijvejin ZA, Golovin K*, et al.* Scalable manufacturing of flexible and highly conductive Ti 3 C 2 T x/PEDOT: PSS thin films for electromagnetic interference shielding. *New J Chem* 2021, **45**(44)**:** 20787-20799.

23. Kamkar M, Ghaffarkhah A, Hosseini E, Amini M, Ghaderi S, Arjmand M. Multilayer Polymeric Nanocomposites for Electromagnetic Interference Shielding: Fabrication, Mechanisms, and Prospects. *New J Chem* 2021.

24. Jaroszewski M, Thomas S, Rane AV. Advanced Materials for Electromagnetic Shielding: Fundamentals, Properties, and Applications. 2018.

25. Aswathi M, Rane AV, Ajitha A, Thomas S, Jaroszewski M. EMI shielding fundamentals. *Advanced Materials for Electromagnetic Shielding: Fundamentals, Properties, and Applications* 2018**:** 1-9.

26. Shen B, Li Y, Zhai W, Zheng W. Compressible graphene-coated polymer foams with ultralow density for adjustable electromagnetic interference (EMI) shielding. *ACS Appl Mater Interfaces* 2016, **8**(12)**:** 8050-8057.

27. Li Q, Chen L, Ding J, Zhang J, Li X, Zheng K*, et al.* Open-cell phenolic carbon foam and electromagnetic interference shielding properties. *Carbon* 2016, **104:** 90-105.

28. Iqbal A, Sambyal P, Koo CM. 2D MXenes for electromagnetic shielding: a review. *Adv Funct Mater* 2020, **30**(47)**:** 2000883.

29. Ott HW. *Electromagnetic compatibility engineering*. John Wiley & Sons, 2011.

30. Han M, Yin X, Hantanasirisakul K, Li X, Iqbal A, Hatter CB*, et al.* Anisotropic MXene aerogels with a mechanically tunable ratio of electromagnetic wave reflection to absorption. *Adv Opt Mater* 2019, **7**(10)**:** 1900267.

31. Lee SH, Yu S, Shahzad F, Kim WN, Park C, Hong SM*, et al.* Density-tunable lightweight polymer composites with dual-functional ability of efficient EMI shielding and heat dissipation. *Nanoscale* 2017, **9**(36)**:** 13432-13440.

32. Anasori B, Gogotsi ÛG. *2D metal carbides and nitrides (MXenes)*. Springer, 2019.

33. Schulz RB, Plantz V, Brush D. Shielding theory and practice. *IEEE Transactions on Electromagnetic Compatibility* 1988, **30**(3)**:** 187-201.

34. Singh AP, Mishra M, Sambyal P, Gupta BK, Singh BP, Chandra A*, et al.* Encapsulation of γ-Fe 2 O 3 decorated reduced graphene oxide in polyaniline core–shell tubes as an exceptional tracker for electromagnetic environmental pollution. *Journal of Materials Chemistry A* 2014, **2**(10)**:** 3581-3593.

35. González M, Pozuelo J, Baselga J. Electromagnetic shielding materials in GHz range. *The Chemical Record* 2018, **18**(7-8)**:** 1000-1009.

36. Kaiser KL. *Electromagnetic shielding*. Crc Press, 2005.

37. Shahzad F, Alhabeb M, Hatter CB, Anasori B, Hong SM, Koo CM*, et al.* Electromagnetic interference shielding with 2D transition metal carbides (MXenes). *Science* 2016, **353**(6304)**:** 1137-1140.

38. Kumar R, Choudhary HK, Pawar SP, Bose S, Sahoo B. Carbon encapsulated nanoscale iron/iron-carbide/graphite particles for EMI shielding and microwave absorption. *Physical Chemistry Chemical Physics* 2017, **19**(34)**:** 23268-23279.

39. Sohi N, Rahaman M, Khastgir D. Dielectric property and electromagnetic interference shielding effectiveness of ethylene vinyl acetate-based conductive composites: Effect of different type of carbon fillers. *Polymer Composites* 2011, **32**(7)**:** 1148-1154.

40. Ghaffarkhah A, Hashemi SA, Rostami S, Amini M, Ahmadijokani F, Pournaghshband Isfahani A*, et al.* Ultra-Flyweight Cryogels of MXene/Graphene Oxide for Electromagnetic Interference Shielding. *Adv Funct Mater* 2023**:** 2304748.

41. Song Q, Ye F, Yin X, Li W, Li H, Liu Y*, et al.* Carbon nanotube–multilayered graphene edge plane core–shell hybrid foams for ultrahigh-performance electromagnetic-interference shielding. *Adv Mater* 2017, **29**(31)**:** 1701583.

42. Zhang J, Kong N, Uzun S, Levitt A, Seyedin S, Lynch PA*, et al.* Scalable Manufacturing of Free-Standing, Strong Ti3C2Tx MXene Films with Outstanding Conductivity. *Adv Mater* 2020**:** 2001093.

43. Zeng J, Ji X, Ma Y, Zhang Z, Wang S, Ren Z*, et al.* 3D graphene fibers grown by thermal chemical vapor deposition. *Adv Mater* 2018, **30**(12)**:** 1705380.

44. Shen B, Zhai W, Zheng W. Ultrathin flexible graphene film: an excellent thermal conducting material with efficient EMI shielding. *Adv Funct Mater* 2014, **24**(28)**:** 4542-4548.

45. Tan M, Chen D, Cheng Y, Sun H, Chen G, Dong S, *et al.* Anisotropically Oriented Carbon Films with Dual-Function of Efficient Heat Dissipation and Excellent Electromagnetic Interference Shielding Performances. *Adv Funct Mater* 2022**:** 2202057.

46. Sun R, Zhang HB, Liu J, Xie X, Yang R, Li Y, *et al.* Highly conductive transition metal carbide/carbonitride (MXene)@ polystyrene nanocomposites fabricated by electrostatic assembly for highly efficient electromagnetic interference shielding. *Adv Funct Mater* 2017, **27**(45)**:** 1702807.

47. Zeng Z, Jin H, Chen M, Li W, Zhou L, Zhang Z. Lightweight and anisotropic porous MWCNT/WPU composites for ultrahigh performance electromagnetic interference shielding. *Adv Funct Mater* 2016, **26**(2)**:** 303-310.

48. Gong S, Sheng X, Li X, Sheng M, Wu H, Lu X, *et al.* A Multifunctional Flexible Composite Film with Excellent Multi-Source Driven Thermal Management, Electromagnetic Interference Shielding, and Fire Safety Performance, Inspired by a "Brick–Mortar" Sandwich Structure. *Adv Funct Mater* 2022**:** 2200570.

49. Chen Y, Zhang HB, Yang Y, Wang M, Cao A, Yu ZZ. High-performance epoxy nanocomposites reinforced with three-dimensional carbon nanotube sponge for electromagnetic interference shielding. *Adv Funct Mater* 2016, **26**(3)**:** 447-455.

50. Li Y, Tian X, Gao SP, Jing L, Li K, Yang H, *et al.* Reversible crumpling of 2D titanium carbide (MXene) nanocoatings for stretchable electromagnetic shielding and wearable wireless communication. *Adv Funct Mater* 2020, **30**(5)**:** 1907451.

51. Zhou Q, Lyu J, Wang G, Robertson M, Qiang Z, Sun B, *et al.* Mechanically strong and multifunctional hybrid hydrogels with ultrahigh electrical conductivity. *Adv Funct Mater* 2021, **31**(40)**:** 2104536.

52. Chen H, Wen Y, Qi Y, Zhao Q, Qu L, Li C. Pristine titanium carbide MXene films with environmentally stable conductivity and superior mechanical strength. *Adv Funct Mater* 2020, **30**(5)**:** 1906996.

53. Ying M, Zhao R, Hu X, Zhang Z, Liu W, Yu J, *et al.* Wrinkled titanium carbide (MXene) with surface charge polarizations through chemical etching for superior electromagnetic interference shielding. *Angew Chem* 2022, **134**(16)**:** e202201323.

54. Ma Z, Xiang X, Shao L, Zhang Y, Gu J. Multifunctional wearable silver nanowire decorated leather nanocomposites for joule heating, electromagnetic interference shielding and piezoresistive sensing. *Angew Chem Int Ed* 2022, **61**(15)**:** e202200705.

55. Shi S, Qian B, Wu X, Sun H, Wang H, Zhang HB*, et al.* Self-Assembly of MXene-Surfactants at Liquid–Liquid Interfaces: From Structured Liquids to 3D Aerogels. *Angew Chem Int Ed* 2019, **58**(50)**:** 18171-18176.

56. Zhang Y, Ruan K, Gu J. Flexible Sandwich-Structured Electromagnetic Interference Shielding Nanocomposite Films with Excellent Thermal Conductivities. *Small* 2021, **17**(42)**:** 2101951.

57. Wan YJ, Zhu PL, Yu SH, Sun R, Wong CP, Liao WH. Anticorrosive, ultralight, and flexible carbon-wrapped metallic nanowire hybrid sponges for highly efficient electromagnetic interference shielding. *Small* 2018, **14**(27)**:** 1800534.

58. Wang L, Qiu H, Liang C, Song P, Han Y, Han Y*, et al.* Electromagnetic interference shielding MWCNT-Fe3O4@ Ag/epoxy nanocomposites with satisfactory thermal conductivity and high thermal stability. *Carbon* 2019, **141:** 506-514.

59. Song W-L, Guan X-T, Fan L-Z, Cao W-Q, Wang C-Y, Zhao Q-L*, et al.* Magnetic and conductive graphene papers toward thin layers of effective electromagnetic shielding. *J Mater Chem A* 2015, **3**(5)**:** 2097-2107.

60. Zhang K, Li G-H, Feng L-M, Wang N, Guo J, Sun K*, et al.* Ultralow percolation threshold and enhanced electromagnetic interference shielding in poly (L-lactide)/multi-walled carbon nanotube nanocomposites with electrically conductive segregated networks. *J Mater Chem C* 2017, **5**(36)**:** 9359-9369.

61. Jia L-C, Li Y-K, Yan D-X. Flexible and efficient electromagnetic interference shielding materials from ground tire rubber. *Carbon* 2017, **121:** 267-273.

62. Sharif F, Arjmand M, Moud AA, Sundararaj U, Roberts EP. Segregated hybrid poly (methyl methacrylate)/graphene/magnetite nanocomposites for electromagnetic interference shielding. *ACS Appl Mater Interfaces* 2017, **9**(16)**:** 14171-14179.

63. Shen B, Li Y, Yi D, Zhai W, Wei X, Zheng W. Microcellular graphene foam for improved broadband electromagnetic interference shielding. *Carbon* 2016, **102:** 154-160.

64. Gebrekrstos A, Biswas S, Menon AV, Madras G, Pötschke P, Bose S. Multi-layered stack consisting of PVDF nanocomposites with flow-induced oriented MWCNT structure can supress electromagnetic radiation. *Compos B Eng* 2019, **166:** 749-757.

65. Zhang H, Zhang G, Tang M, Zhou L, Li J, Fan X*, et al.* Synergistic effect of carbon nanotube and graphene nanoplates on the mechanical, electrical and electromagnetic interference shielding properties of polymer composites and polymer composite foams. *Chem Eng J* 2018, **353:** 381-393.

66. Kong L, Yin X, Xu H, Yuan X, Wang T, Xu Z, *et al.* Powerful absorbing and lightweight electromagnetic shielding CNTs/RGO composite. *Carbon* 2019, **145:** 61-66.

67. Sheng A, Ren W, Yang Y, Yan D-X, Duan H, Zhao G, *et al.* Multilayer WPU conductive composites with controllable electro-magnetic gradient for absorption-dominated electromagnetic interference shielding. *Compos - A: Appl Sci Manuf* 2020, **129:** 105692.

68. Zhou B, Zhang Z, Li Y, Han G, Feng Y, Wang B, *et al.* Flexible, robust, and multifunctional electromagnetic interference shielding film with alternating cellulose nanofiber and MXene layers. *ACS Appl Mater Interfaces* 2020, **12**(4)**:** 4895-4905.

69. Xu H, Yin X, Li X, Li M, Liang S, Zhang L, *et al.* Lightweight Ti2CT x MXene/poly (vinyl alcohol) composite foams for electromagnetic wave shielding with absorption-dominated feature. *ACS Appl Mater Interfaces* 2019, **11**(10)**:** 10198-10207.

70. Yang G-Y, Wang S-Z, Sun H-T, Yao X-M, Li C-B, Li Y-J, *et al.* Ultralight, Conductive Ti3C2T x MXene/PEDOT: PSS Hybrid Aerogels for Electromagnetic Interference Shielding Dominated by the Absorption Mechanism. *ACS Appl Mater Interfaces* 2021, **13**(48)**:** 57521-57531.

71. Yin G, Wang Y, Wang W, Yu D. Multilayer structured PANI/MXene/CF fabric for electromagnetic interference shielding constructed by layer-by-layer strategy. *Colloids and Surfaces A: Physicochemical and Engineering Aspects* 2020, **601:** 125047.

72. Jin X, Wang J, Dai L, Liu X, Li L, Yang Y, *et al.* Flame-retardant poly (vinyl alcohol)/MXene multilayered films with outstanding electromagnetic interference shielding and thermal conductive performances. *Chem Eng J* 2020, **380:** 122475.

73. Lu J, Cheng L, Liao C, Jia P, Song L, Wang B, *et al.* Ultrathin and Mechanically Robust Mussel Byssus-Inspired MXene@ Aramid Nanofibers Materials with Superior Endurance in Harsh Environments for Tunable EMI Shielding Performance. *Adv Mater Interfaces* 2022**:** 2101359.

74. Habibpour S, Zarshenas K, Zhang M, Hamidinejad M, Ma L, Park CB, *et al.* Greatly Enhanced Electromagnetic Interference Shielding Effectiveness and Mechanical Properties of Polyaniline-Grafted Ti3C2T x MXene–PVDF Composites. *ACS Appl Mater Interfaces* 2022.

75. Zeng Z, Jiang F, Yue Y, Han D, Lin L, Zhao S, *et al.* Flexible and ultrathin waterproof cellular membranes based on high-conjunction metal-wrapped polymer nanofibers for electromagnetic interference shielding. *Adv Mater* 2020, **32**(19)**:** 1908496.

76. Xie Y, Liu S, Huang K, Chen B, Shi P, Chen Z, *et al.* Ultra-broadband Strong Electromagnetic Interference Shielding with Ferromagnetic Graphene Quartz Fabric. *Adv Mater* 2022**:** 2202982.

77. Wei Q, Pei S, Qian X, Liu H, Liu Z, Zhang W*, et al.* Superhigh electromagnetic interference shielding of ultrathin aligned pristine graphene nanosheets film. *Adv Mater* 2020, **32**(14)**:** 1907411.

78. Liu J, Mckeon L, Garcia J, Pinilla S, Barwich S, Möbius M*, et al.* Additive Manufacturing of Ti3C2-MXene-Functionalized Conductive Polymer Hydrogels for Electromagnetic-Interference Shielding. *Adv Mater* 2022, **34**(5)**:** 2106253.

79. Huang M, Wang L, Li X, Wu Z, Zhao B, Pei K*, et al.* Magnetic Interacted Interaction Effect in MXene Skeleton: Enhanced Thermal-Generation for Electromagnetic Interference Shielding. *Small* 2022**:** 2201587.

80. Wang J, Li Q, Li K, Sun X, Wang Y, Zhuang T*, et al.* Ultra-high electrical conductivity in filler-free polymeric hydrogels toward thermoelectrics and electromagnetic interference shielding. *Advanced materials (Deerfield Beach, Fla)* 2022, **34**(12)**:** e2109904.

81. Wu H, Xie Y, Ma Y, Zhang B, Xia B, Zhang P*, et al.* Aqueous MXene/Xanthan Gum Hybrid Inks for Screen-Printing Electromagnetic Shielding, Joule Heater, and Piezoresistive Sensor. *Small* 2022, **18**(16)**:** 2107087.

82. Wu S, Zou M, Li Z, Chen D, Zhang H, Yuan Y*, et al.* Robust and stable Cu nanowire@ graphene core–shell aerogels for ultraeffective electromagnetic interference shielding. *Small* 2018, **14**(23)**:** 1800634.

83. Zeng Z, Jin H, Chen M, Li W, Zhou L, Xue X*, et al.* Microstructure design of lightweight, flexible, and high electromagnetic shielding porous multiwalled carbon nanotube/polymer composites. *Small* 2017, **13**(34)**:** 1701388.

84. Huang X, Huang J, Zhou G, Wei Y, Wu P, Dong A*, et al.* Gelation-Assisted Assembly of Large-Area, Highly Aligned, and Environmentally Stable MXene Films with an Excellent Trade-Off between Mechanical and Electrical Properties. *Small* 2022**:** 2200829.

85. Han M, Shuck CE, Rakhmanov R, Parchment D, Anasori B, Koo CM*, et al.* Beyond Ti3C2T x: MXenes for Electromagnetic Interference Shielding. *ACS nano* 2020, **14**(4)**:** 5008-5016.

86. Xu J, Li R, Ji S, Zhao B, Cui T, Tan X*, et al.* Multifunctional graphene microstructures inspired by honeycomb for ultrahigh performance electromagnetic interference shielding and wearable applications. *ACS nano* 2021, **15**(5)**:** 8907-8918.

87. Choi HK, Lee A, Park M, Lee DS, Bae S, Lee S-K*, et al.* Hierarchical porous film with layer-by-layer assembly of 2D copper nanosheets for ultimate electromagnetic interference shielding. *ACS nano* 2021, **15**(1)**:** 829-839.

88. Zeng Z, Wu T, Han D, Ren Q, Siqueira G, Nyström G. Ultralight, flexible, and biomimetic nanocellulose/silver nanowire aerogels for electromagnetic interference shielding. *Acs Nano* 2020, **14**(3)**:** 2927-2938.

89. Hu P, Lyu J, Fu C, Gong W-b, Liao J, Lu W*, et al.* Multifunctional aramid nanofiber/carbon nanotube hybrid aerogel films. *ACS nano* 2019, **14**(1)**:** 688-697.

90. Tan X, Liu T-H, Zhou W, Yuan Q, Ying J, Yan Q*, et al.* Enhanced Electromagnetic Shielding and Thermal Conductive Properties of Polyolefin Composites with a Ti3C2T x MXene/Graphene Framework Connected by a Hydrogen-Bonded Interface. *ACS nano* 2022.

91. Cao W-T, Chen F-F, Zhu Y-J, Zhang Y-G, Jiang Y-Y, Ma M-G*, et al.* Binary strengthening and toughening of MXene/cellulose nanofiber composite paper with nacre-inspired structure and superior electromagnetic interference shielding properties. *Acs Nano* 2018, **12**(5)**:** 4583-4593.

92. Lee GS, Yun T, Kim H, Kim IH, Choi J, Lee SH*, et al.* Mussel Inspired Highly Aligned Ti3C2T x MXene Film with Synergistic Enhancement of Mechanical Strength and Ambient Stability. *ACS nano* 2020, **14**(9)**:** 11722-11732.

93. Zhou T, Xu C, Liu H, Wei Q, Wang H, Zhang J*, et al.* Second time-scale synthesis of high-quality graphite films by quenching for effective electromagnetic interference shielding. *ACS nano* 2020, **14**(3)**:** 3121-3128.

94. He X, Feng L, Zhang Z, Hou X, Ye X, Song Q*, et al.* High-performance multifunctional carbon–silicon carbide composites with strengthened reduced graphene oxide. *ACS nano* 2021, **15**(2)**:** 2880-2892.

95. Yang Y, Chen S, Li W, Li P, Ma J, Li B*, et al.* Reduced graphene oxide conformally wrapped silver nanowire networks for flexible transparent heating and electromagnetic interference shielding. *Acs Nano* 2020, **14**(7)**:** 8754-8765.

96. Zhao S, Zhang H-B, Luo J-Q, Wang Q-W, Xu B, Hong S*, et al.* Highly electrically conductive three-dimensional Ti3C2T x MXene/reduced graphene oxide hybrid aerogels with excellent electromagnetic interference shielding performances. *ACS nano* 2018, **12**(11)**:** 11193-11202.

97. Fu C, Sheng Z, Zhang X. Laminated Structural Engineering Strategy toward Carbon Nanotube-Based Aerogel Films. *ACS nano* 2022.

98. Wang J, Ma X, Zhou J, Du F, Teng C. Bioinspired, High-Strength, and Flexible MXene/Aramid Fiber for Electromagnetic Interference Shielding Papers with Joule Heating Performance. *ACS nano* 2022, **16**(4)**:** 6700-6711.

99. Wan Y-J, Wang X-Y, Li X-M, Liao S-Y, Lin Z-Q, Hu Y-G*, et al.* Ultrathin densified carbon nanotube film with “metal-like” conductivity, superior mechanical strength, and ultrahigh electromagnetic interference shielding effectiveness. *ACS nano* 2020, **14**(10)**:** 14134-14145.

100. Li Y, Liu J, Wang E, Shen B, Chen J, Zhang M*, et al.* Controllable growth of NiCo compounds with different morphologies and structures on carbon fabrics as EMI shields with improved absorptivity. *Carbon* 2022, **197:** 508-518.

101. Wu Z, Dong J, Li X, Zhao X, Ji C, Zhang Q. Interlayer decoration of expanded graphite by polyimide resins for preparing highly thermally conductive composites with superior electromagnetic shielding performance. *Carbon* 2022.

102. Wang K, Chen C, Zheng Q, Xiong J, Liu H, Yang L*, et al.* Multifunctional recycled carbon fiber-Ti3C2Tx MXene paper with superior electromagnetic interference shielding and photo/electro-thermal conversion performances. *Carbon* 2022, **197:** 87-97.

103. Zhao B, Bai P, Yuan M, Yan Z, Fan B, Zhang R*, et al.* Recyclable magnetic carbon foams possessing voltage-controllable electromagnetic shielding and oil/water separation. *Carbon* 2022.

104. Yang C, Liu W, Liu N, Su J, Li L, Xiong L*, et al.* Graphene aerogel broken to fragments for a piezoresistive pressure sensor with a higher sensitivity. *ACS Appl Mater Interfaces* 2019, **11**(36)**:** 33165-33172.

105. Huang J, Liu X, Yang Z, Wu X, Wang J, Yang S. Extremely elastic and conductive N-doped graphene sponge for monitoring human motions. *Nanoscale* 2019, **11**(3)**:** 1159-1168.

106. Wang T, Li J, Zhang Y, Liu F, Zhang B, Wang Y*, et al.* Highly ordered 3D porous graphene sponge for wearable piezoresistive pressure sensor applications. *Chemistry–A European Journal* 2019, **25**(25)**:** 6378-6384.

107. Zhu C, Han T, Duoss EB, Golobic AM, Kuntz JD, Spadaccini CM*, et al.* Highly compressible 3D periodic graphene aerogel microlattices. *Nat Commun* 2015, **6**(1)**:** 1-8.

108. Yang M, Zhao N, Cui Y, Gao W, Zhao Q, Gao C*, et al.* Biomimetic architectured graphene aerogel with exceptional strength and resilience. *ACS nano* 2017, **11**(7)**:** 6817-6824.

109. Kim SJ, Mondal S, Min BK, Choi C-G. Highly sensitive and flexible strain–pressure sensors with cracked paddy-shaped MoS2/graphene foam/Ecoflex hybrid nanostructures. *ACS Appl Mater Interfaces* 2018, **10**(42)**:** 36377-36384.

110. Yao HB, Ge J, Wang CF, Wang X, Hu W, Zheng ZJ*, et al.* A flexible and highly pressure-sensitive graphene–polyurethane sponge based on fractured microstructure design. *Adv Mater* 2013, **25**(46)**:** 6692-6698.

111. Luo N, Huang Y, Liu J, Chen SC, Wong CP, Zhao N. Hollow-structured graphene–silicone-composite-based piezoresistive sensors: Decoupled property tuning and bending reliability. *Adv Mater* 2017, **29**(40)**:** 1702675.

112. Tao L-Q, Zhang K-N, Tian H, Liu Y, Wang D-Y, Chen Y-Q*, et al.* Graphene-paper pressure sensor for detecting human motions. *ACS nano* 2017, **11**(9)**:** 8790-8795.

113. Chen Z, Zhuo H, Hu Y, Lai H, Liu L, Zhong L*, et al.* Wood-derived lightweight and elastic carbon aerogel for pressure sensing and energy storage. *Adv Funct Mater* 2020, **30**(17)**:** 1910292.

114. Zhu M, Yue Y, Cheng Y, Zhang Y, Su J, Long F*, et al.* Hollow MXene sphere/reduced graphene aerogel composites for piezoresistive sensor with ultra-high sensitivity. *Adv Electron Mater* 2020, **6**(2)**:** 1901064.

115. Zhuo H, Hu Y, Chen Z, Peng X, Liu L, Luo Q*, et al.* A carbon aerogel with super mechanical and sensing performances for wearable piezoresistive sensors. *J Mater Chem A* 2019, **7**(14)**:** 8092-8100.

116. Yang Z, Li H, Zhang S, Lai X, Zeng X. Superhydrophobic MXene@ carboxylated carbon nanotubes/carboxymethyl chitosan aerogel for piezoresistive pressure sensor. *Chem Eng J* 2021, **425:** 130462.

117. Qin L, Yang D, Zhang M, Zhao T, Luo Z, Yu Z-Z. Superelastic and ultralight electrospun carbon nanofiber/MXene hybrid aerogels with anisotropic microchannels for pressure sensing and energy storage. *J Colloid Interface Sci* 2021, **589:** 264-274.

118. Yang G, Yang Y, Chen T, Wang J, Ma L, Yang S. Graphene/MXene Composite Aerogels Reinforced by Polyimide for Pressure Sensing. *ACS Applied Nano Materials* 2022, **5**(1)**:** 1068-1077.

119. Liu H, Chen X, Zheng Y, Zhang D, Zhao Y, Wang C*, et al.* Lightweight, superelastic, and hydrophobic polyimide nanofiber/MXene composite aerogel for wearable piezoresistive sensor and oil/water separation applications. *Adv Funct Mater* 2021, **31**(13)**:** 2008006.

120. Ma Y, Yue Y, Zhang H, Cheng F, Zhao W, Rao J*, et al.* 3D synergistical MXene/reduced graphene oxide aerogel for a piezoresistive sensor. *Acs Nano* 2018, **12**(4)**:** 3209-3216.

121. Tian Y, Han J, Yang J, Wu H, Bai H. A highly sensitive graphene aerogel pressure sensor inspired by fluffy spider leg. *Adv Mater Interfaces* 2021, **8**(15)**:** 2100511.

122. Long S, Feng Y, He F, Zhao J, Bai T, Lin H*, et al.* Biomass-derived, multifunctional and wave-layered carbon aerogels toward wearable pressure sensors, supercapacitors and triboelectric nanogenerators. *Nano Energy* 2021, **85:** 105973.
