# Supplementary material for: Functional Janus structured liquids and aerogels
Source: Nat Commun. 2023 Nov 28;14:7811. doi: 10.1038/s41467-023-43319-7 (PMC10684591; doi:10.1038/s41467-023-43319-7)
Supplement: Supplementary file 3 — Description of Additional Supplementary Files [file 41467_2023_43319_MOESM3_ESM.docx]

**Legends for Supplementary Videos**

Supplementary Video S1: Formation of non-responsive liquid threads of GO.

Supplementary Video S2: Mechanical stability of GO liquid threads.

Supplementary Video S3: Response of mGO/GO liquid threads to an external magnetic field.

Supplementary Video S4: Formation of Ti_3_C_2_T_x_ liquid beads.

Supplementary Video S5: Formation of conductive liquid threads of Ti_3_C_2_T_x_/GO.

Supplementary Video S6: Formation of magnetic/conductive Janus liquid threads.

Supplementary Video S7: Formation of non-responsive/conductive Janus liquid threads.

Supplementary Video S8: Response of magnetic/conductive Janus aerogel to an external magnetic field.

Supplementary Video S9: Ti_3_C_2_T_x_/GO worm-like aerogels have no elastic recovery after compression.

Supplementary Video S10: Worm-like aerogels of pure GO with elastic behavior.

Supplementary Video S11: Elastic behavior of Janus aerogels.

Supplementary Video S12: Janus aerogels are mechanically stable and can be easily handled.

Supplementary Video S13: The recorded signal for the soft press of the Janus sensor.

Supplementary Video S14: The recorded signal for the hard press of the Janus sensor.

Supplementary Video S15: The recorded signal for the soft press of the Janus sensor, i.e., three months after its fabrication.

Supplementary Video S16: The capability of the fabricated device to distinguish different letters and even polysyllabic words when attached to the skin outside of the larynx of a human subject.
